# Smokefree legislation effects on respiratory and sensory disorders: A systematic review and meta-analysis

**DOI:** 10.1371/journal.pone.0181035

**Published:** 2017-07-31

**Authors:** Yolanda Rando-Matos, Mariona Pons-Vigués, María José López, Rodrigo Córdoba, José Luis Ballve-Moreno, Elisa Puigdomènech-Puig, Vega Estíbaliz Benito-López, Olga Lucía Arias-Agudelo, Mercè López-Grau, Anna Guardia-Riera, José Manuel Trujillo, Carlos Martin-Cantera

**Affiliations:** 1 Centre d'Atenció Primària (CAP) Florida Nord. Gerència d’Àmbit d’Atenció Primària Metropolitana Sud, Institut Català de la Salut (ICS), Hospitalet de Llobregat, Barcelona, Spain; 2 Institut Universitari d’Investigació en Atenció Primària Jordi Gol (IDIAP Jordi Gol), Barcelona, Spain; 3 Universitat Autònoma de Barcelona, Bellaterra (Cerdanyola del Vallès), Spain; 4 Universitat de Girona, Girona, Spain; 5 Public Health Agency of Barcelona, Barcelona, Spain; 6 CIBER de Epidemiología y Salud Pública (CIBERESP), Barcelona, Spain; 7 Institut d'Investigació Biomèdic (IIB Sant Pau), Barcelona, Spain; 8 Universitat Pompeu Fabra (UPF), Barcelona, Spain; 9 Centro de Salud Universitario Delicias Sur, Servicio Aragonés de Salud (SALUD), Zaragoza, Spain; 10 Universidad de Zaragoza, Zaragoza, Spain; 11 Agència de Qualitat i Avaluació Sanitàries, AQuAS, Generalitat de Catalunya, Barcelona, Spain; 12 Servicio de Medicina Preventiva, Complejo Asistencial Universitario de Salamanca, Sanidad de Castilla y Leon (SACYL), Salamanca, Spain; 13 Grupo de investigación: Trastornos sensoriales y neuroplasticidad cerebral (UIC: 083), Instituto de Investigación Biomédica de Salamanca (IBSAL), Salamanca, Spain; 14 Instituto de Neurociencias de Castilla y León (INCYL), Salamanca, Spain; 15 Centre d'Atenció Primària (CAP) San Martí de Provençals, Gerència d’Àmbit d’Atenció Primària Barcelona Ciutat, Institut Català de la Salut (ICS), Barcelona, Spain; 16 Centre d'Atenció Primària (CAP) Passeig de Sant Joan, Gerència d’Àmbit d’Atenció Primària Barcelona Ciutat, Institut Català de la Salut (ICS), Barcelona, Spain; 17 Àrea Bàsica de Salut l'Hospitalet de Llobregat 6—Sta. Eulàlia sud, Gerència d’Àmbit d’Atenció Primària Hospitalet de Llobregat, Institut Català de la Salut, Barcelona, Spain; 18 Centro de Salud Cuevas del Almanzora, Almería, Spain; Telethon Institute for Child Health Research, AUSTRALIA

## Abstract

**Aims:**

The aim of this systematic review and meta-analysis is to synthesize the available evidence in scientific papers of smokefree legislation effects on respiratory diseases and sensory and respiratory symptoms (cough, phlegm, red eyes, runny nose) among all populations.

**Materials and methods:**

Systematic review and meta-analysis were carried out. A search between January 1995 and February 2015 was performed in PubMed, EMBASE, Cochrane Library, Scopus, Web of Science, and Google Scholar databases. Inclusion criteria were: 1) original scientific studies about smokefree legislation, 2) Data before and after legislation were collected, and 3) Impact on respiratory and sensory outcomes were assessed. Paired reviewers independently carried out the screening of titles and abstracts, data extraction from full-text articles, and methodological quality assessment.

**Results:**

A total number of 1606 papers were identified. 50 papers were selected, 26 were related to symptoms (23 concerned workers). Most outcomes presented significant decreases in the percentage of people suffering from them, especially in locations with comprehensive measures and during the immediate post-ban period (within the first six months). Four (50%) of the papers concerning pulmonary function reported some significant improvement in expiratory parameters. Significant decreases were described in 13 of the 17 papers evaluating asthma hospital admissions, and there were fewer significant reductions in chronic obstructive pulmonary disease admissions (range 1–36%) than for asthma (5–31%). Six studies regarding different respiratory diseases showed discrepant results, and four papers about mortality reported significant declines in subgroups. Low bias risk was present in 23 (46%) of the studies.

**Conclusions:**

Smokefree legislation appears to improve respiratory and sensory symptoms at short term in workers (the overall effect being greater in comprehensive smokefree legislation in sensory symptoms) and, to a lesser degree, rates of hospitalization for asthma.

## Introduction

Passive exposure to tobacco smoke (also known as exposure to environmental tobacco smoke, second-hand smoke, and passive smoking) multiplies the risk of coronary disease and lung cancer in adults. It also exacerbates asthma and respiratory symptoms, and increases the risk of sudden infant death syndrome amongst other health effects[1]. All of the above has led to legislative measures being adopted in order to protect the population’s health in public areas and workplaces[2]. In 1998, in the United States, California was the first to put into practice these measures[3,4], and from 2004 all the members of the European Union have adopted some kind of regulation[5]. There are different types of smokefree legislation (SFL): comprehensive (smoking is prohibited in all closed public areas and workplaces including public transport, bars, and restaurants) and partial (smoking is allowed in some private workplaces, for instance in the hospitality and entertainment sectors)[6]. Numerous studies have been published evaluating the impact of SFL from different perspectives: reduction of exposure to second-hand smoke[7], prevalence of tobacco use (no consistent evidence of a reduction attributable to SFL)[8], cardiovascular mortality (studies related to cardiovascular mortality have conflicting results possibly due to the quality of some of these papers)[9–12], cardiovascular morbidity (consistent evidence of reductions in cardiac events and hospitalizations following implementation of SFL)[13–19], and economic impact (SFL does not adversely affect business revenues or operating costs)[20] amongst others. Most systematic reviews have evaluated cardiovascular effects[15–19], tobacco consumption[8], exposure to second-hand smoke[7], and, in a more heterogeneous manner, respiratory diseases at population levels[8,21]. This last issue is, to the best of our knowledge, the least studied field.

A meta-analysis concerning the impact of banning smoking in the workplace concluded that the measure protected non-smokers from passive exposure and encouraged smokers to reduce their consumption[22]. A Cochrane Review from 2010 with 12 studies found that in ten of them legislation decreased respiratory symptoms in workers, some of the studies only assessed non-smokers[8]. A review of the impact of SFL on health and economic outcomes[20] concluded that there is a need for further research on indicators such as asthma and chronic obstructive pulmonary disease (COPD) with special attention regarding low-income populations, women, racial/ethnic minorities, and older adults. Tan and Glantz observed in a meta-analysis (2012) a 24% decrease in hospital admissions due to respiratory disease[23]. However, an updated Cochrane Review (2016) with the same inclusion criteria as the first review determined that effects of SFL on respiratory health, including COPD, asthma, and lung function were inconsistent[21].

It is essential to evaluate the impact of SFL on health[24]. In addition, a systematic review of the influence of laws which ban smoking in certain places on respiratory problems (e.g. asthma, COPD, upper and lower respiratory tract infections, and even ear, nose, and throat diseases), and in populations of all ages, irrespective of presenting respiratory diseases, would add greater value to the evidence obtained in other fields. To the best of our knowledge there is no overall, systematic review concerning the effect of SFL on respiratory and sensory symptomatology and disease. For a first approach, gathering data from previous reviews would be of great interest even if the populations differed.

The aim of this study is to synthesize the available evidence published in scientific journals on the effects of SFL on respiratory and sensory symptoms and diseases among all populations. Specifically, the objective is to assess the effects of SFL on admissions and emergency visits at hospital/primary health care centers, treatment use, and mortality in individuals suffering from asthma, COPD, and other respiratory diseases.

## Materials and methods

### Data sources

A systematic review and meta-analysis were carried out to select published papers that assessed respiratory and sensory effects of SFL according to the Preferred Reporting Items for Systematic Reviews and Meta Analysis (PRISMA) guidelines[25]. The study protocol (CRD42015019647) was published in the International Prospective Register of Systematic Reviews (PROSPERO) (http://www.crd.york.ac.uk/PROSPERO/display_record.asp?ID=CRD42015019647).

Searches were conducted in: PubMed, EMBASE, Cochrane Library, Scopus, Web of Science, and Google Scholar. S1 Table illustrates the different strategies. Due to the fact that a preliminary Google Scholar search revealed a considerable number of hits, only the first 200 results were scanned as prior bibliography[26–28]. This figure ensures the most relevant articles were obtained[29] and increased the sensitivity of the search[30]. A manual search in the reference lists of systematic reviews was performed. In addition, a national expert in tobacco control and public health was contacted to identify additional studies that might not have been obtained in the process. These sources were combined in order to gather as many relevant data as possible.

Inclusion criteria were: 1) Information concerning SFL (comprehensive or partial) implemented at national, regional, local, and workplace level; 2) Evaluation of SFL with data before and after its implementation; 3) Assessment of the impact of SFL on respiratory diseases (e.g. asthma, COPD and other pulmonary diseases, upper and lower respiratory tract infections, and ear, nose, and throat diseases) and outcomes including hospitalization and mortality/morbidity rate, respiratory (any respiratory symptom, wheezing, phlegm, morning cough, cough during the rest of the day, breathlessness, tightness in chest and asthma symptoms) and sensory symptoms (any sensory symptom, red/irritated eyes, runny nose/sneezing and sore/scratchy throat); 4) All populations irrespective of age, health or smoking status; 5) Original papers in peer-reviewed journals published from January 1, 1995. This date was selected as it is considered to be the first year a more comprehensive legislation was established with respect to workplaces and restaurants[6]; and 6) Papers written in English, French, Portuguese, Italian, Catalan or Spanish. Exclusion criteria concerning type of study design were editorials, letters to the editor, systematic reviews, conference proceedings, cost studies, and theoretical papers.

### Study selection

**Fig 1:**
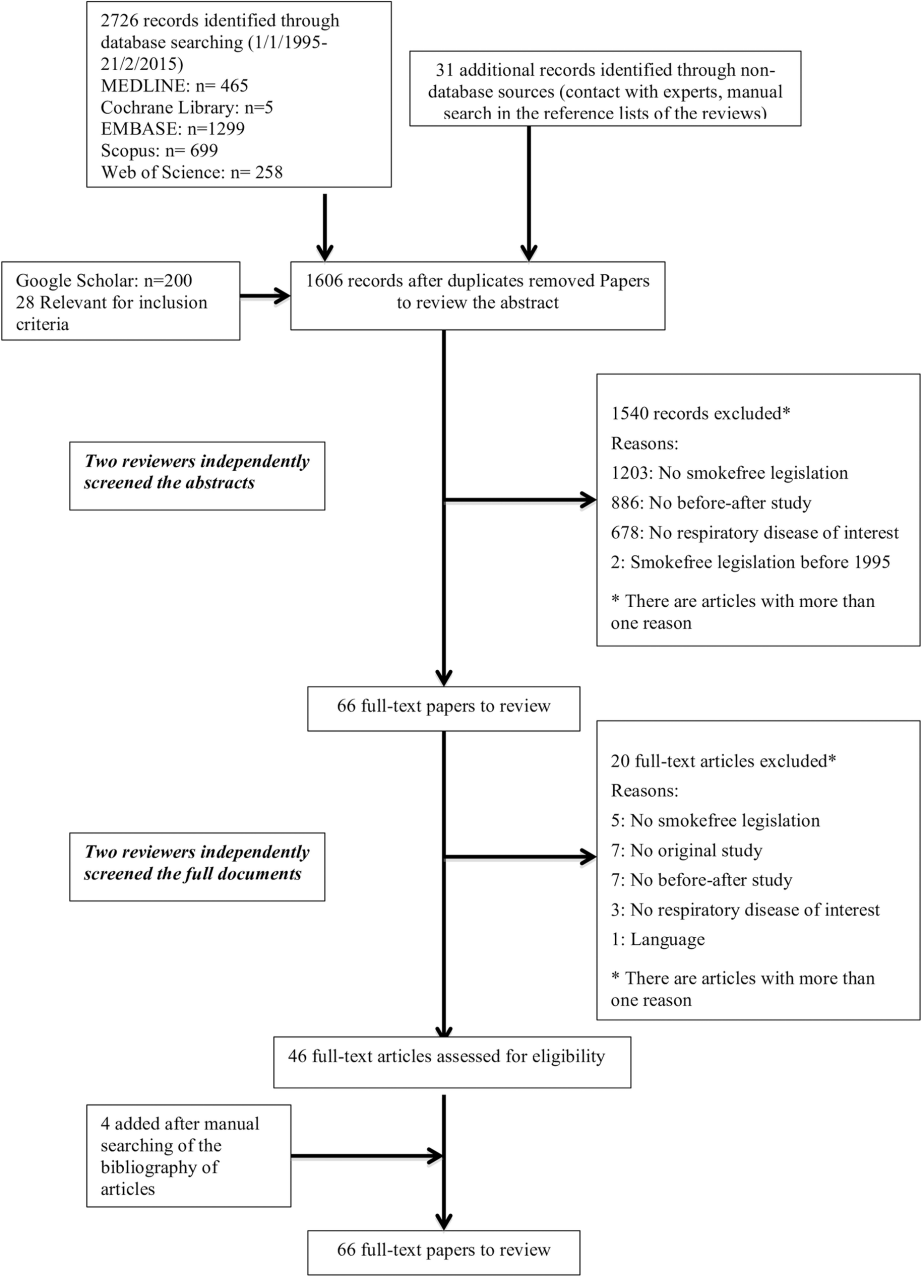
Flow diagram of literature search and study selection from papers evaluating smokefree legislation effects on respiratory and sensory disorders (1995–2015).

Study selection was composed of various stages (Fig 1): First, in February, 2015, the searches were carried out by two authors who identified 2726 papers. Thirty-one additional records were found through non database sources (contact with experts and manual search in the reference lists of the reviews) and 28 from Google Scholar. After removing duplicates 1606 records remained. Second, 12 paired researchers independently screened the titles and abstracts to see whether the papers met the inclusion criteria. Motives for exclusion were recorded. There were two additional reviewers for discrepancies. 1540 records were excluded leaving 66 full-text papers to review. Third, the reviewers read the 66 full-text papers, 46 of which were included in the study and four additional ones were accepted after manual exploration of the bibliography. Finally, 50 papers were selected[31–80] for data extraction and quality assessment.

### Data extraction and quality assessment

The following data were extracted from each full-text paper: Reference (author, year of publication), country, region, aims, type of legislation (i.e. comprehensive SFL banning smoking in virtually all indoor workplaces and public areas, including bars, restaurants and public transport with no designated smoking areas permitted, and partial which covers fewer locations[4,6,81]), study period, study design (following the classification of “Evaluative Designs in Public health: Methodological Considerations” [82] which groups the main evaluation designs in public health as non-experimental and quasi-experimental, and within each of these there are before-after studies and temporal series), study participants, sample size, respiratory and sensory outcomes, source of information, summary of findings, competing interest, and risk of bias (RoB). The latter was evaluated by a version we adapted of the Suggested Risk of Bias Criteria for the Cochrane Effective Practice and Organization of Care Group[83] (EPOC) reviews for uncontrolled before-and-after studies and interrupted time series. We included some domains/subdomains and some other were discarded others due to their unsuitability for evaluation studies and for the purpose of assessing non-randomized and non-comparative studies as in prior bibliography[84]. Overall methodological quality was rated as low, moderate, or high RoB (S2 Table). Revision of titles and abstracts, data extraction from full-text papers, and quality assessment were performed by two authors (six pairs of reviewers) independently and checked by a third in the case of discrepancies.

### Data analysis

Data were synthesized through a narrative review with summary and quantitative descriptive analysis. IBM SPSS Statistics V21.0 was employed for the descriptive analysis. A meta-analysis was performed using Review Manager (RevMan, version 5.3). The effect of SFL was estimated by mean differences (MD) in continuous outcomes and risk ratios (RR) and risk difference (RD) in dichotomous ones. Pooled effect measures were computed applying the inverse-variance method in a random-effect model. Heterogeneity was quantified with the I2 statistic, which describes the proportion of the total between-study variability due to heterogeneity[85]. If the P-value was less than 0.10 and I2 exceeded 50%, heterogeneity was considered to be substantial. Subgroup analysis was used to evaluate whether results differed according to the outcome (respiratory symptoms, sensory symptoms, spirometry parameters, and asthma, COPD and pneumonia/bronchitis admissions), type of SFL (comprehensive vs partial) and population (general population vs adult vs children). Within these subgroups, further subdivisions by study design (non experimental vs quasi-experimental), the quality of included studies (high vs moderate vs low RoB), and follow-up time (< or ≥12 months in the case of outcomes referred to symptomatology and < or ≥24 months in those referred to pathology) were carried out. When statistical heterogeneity was detected and there were more than three studies involved, several sensitivity analysis were performed assessing the relative influence of each study on pooled estimates by omitting one study at a time. Further reduced sets of analyzes were continued successively until I2 dropped below the intended threshold 50% (no more than two studies were drawn). Publication bias was assessed by using funnel plots if the meta-analysis included at least ten studies[86].

## Results

### Characteristics of studies

A total number of 1606 papers was identified. Information for each paper is presented in Tables 1, 2 and 3 and papers are classified by type of outcomes. S3 Table shows the characteristics of the 50 included studies[31–80]. The United States was the country with most publications (16 papers, 32.0%), and 24 (48.0%) collectively came from Europe. The hospitality sector represented 44.0% of the studies (22 papers, same percentage for city and regional locations), 33 (66.0%) evaluated comprehensive SFL, 27 (54.0%) presented a non-experimental before-after design (without control group), and 25 (50.0%) had a study population comprising of hospitality workers. The papers that assessed the effect of SFL on lung symptomatology and function had follow-up periods from one month to two years. In contrast, those evaluating diseases had longer periods: from eleven months to seven years. The most evaluated outcomes were respiratory (26 papers, 52.0%; symptoms were any respiratory symptom, wheezing, phlegm, morning cough, cough during the rest of the day, breathlessness, tightness in chest, and asthma symptoms) and sensory (19 papers, 38.0%; symptoms were any sensory symptom, red/irritated eyes, runny nose/sneezing and sore/scratchy throat) symptomatology followed by hospital admissions for asthma (17 papers, 34.0%) and COPD (nine papers, 18.0%).

**Table 1:** Summary of studies on the impact of smoke-free legislation on symptomatology.

**Respiratory symptoms**								
**Aims**	**Legislation**	**Study period**	**Study design**	**Study participants and size**	**Variables**	**Source of information**	**Summary of findings**	**Risk of Bias**
**Allwright, 2005. The Republic and Northern Ireland** (three areas in the Republic-Dublin, Cork, and County Galway- intervention- and one area in Northern Ireland -control) [31]								
To compare respiratory health in bar staff in rural and urban areas of the Republic of Ireland before and after the law and to compare these changes with changes observed in Northern Ireland.	Comprehensive. *Ban*: March 2004	*Pre-ban*: September 2003 *Post-ban*: March 2005	Pre-posttest quasi-experimental design	226 participants in the baseline and 213 in the follow-up survey. Of these, 158 were non-smokers. Republic Ireland: Mean age 45.5, 17% women. Northern Ireland: Mean age 36.1, 25% women.	Any symptom, wheezing/whistlig, shortness of breath cough, morning cough, cough during the rest, production of phlegm in non-smokers	International Union Against Tuberculosis and Lung Disease (IUATLD) Bronchial Symptoms Questionnaire.	At baseline 65% of non-smokers in the Republic reported one or more respiratory symptom. This dropped by 25% to 49% (p 0.001) at follow-up. After the ban, fewer reported cough during the day or night (p 0.004) or production of phlegm (p 0.002). In Northern Ireland, the proportion reporting any respiratory symptom was lower at baseline (45%) than in the Republic and remained at 45% after the ban. The adjusted rate ratio for the number of respiratory symptoms in the Republic dropped (from 1.33 to 0.98), while in Northern Ireland it increased by 16% (from 0.67 to 0.83).	Low
**Ayres, 2009. Scotland** [32]								
To examine changes in prevalence of self-reported respiratory and sensory symptoms of bar workers after smoke-free legislation (SFL) was introduced	Comprehensive *Ban*: 26 March 2006	*Pre-ban*: 7 Jan 2006 *Post-ban*: May- July 2006 and January—March 2007	Pre-posttest non-experimental design	371 bar workers, including managers, owners and bar staff non smokers, smokers and ex-smokers. Only 177 at 3 phases. Mean age 29.5, male 51%	Self-reported wheeze, shortness of breath, morning cough, other cough, phlegm, any respiratory symptom.	IUATLD Bronchial Symptoms Questionnaire	Of the 191 (51%) workers seen at 1-year follow- up, any respiratory symptom fell from 69% to 57% (p 0.02), effects being greater at 2 months. The reduction in respiratory symptoms was similar although greater for ‘‘any” sensory symptom (69% falling to 54%, p 0.011). For non-smokers (n = 57) the reductions in reported symptoms were significant for phlegm production (32% to 14%). Wheeze (48% to 31%) and breathlessness (42% to 29%) improved significantly in smokers.	Low
**Bannon 2009, North of Ireland.** Belfast [40]								
To assess, before and after the introduction of the SFL, bar workers’ self-reported levels of respiratory symptoms	Comprehensive *Ban*: April 2007	*Pre-ban*: March 2007 *Post-ban*: July 2007	Pre-posttest non-experimental design	97 (pre-ban) and101 (post- ban) bar workers of the 35 Belfast bars. Number of female before 39, after 42; Male before 58, after 59. The majority rank of age: 16–25 and 26–35 years	Wheeze/whistling in the chest, shortness of breath, cough first thing in the morning, cough in day or night, at least one respiratory symptoms.	A short self-completing questionnaire similar to IUATLD Bronchial Symptoms Questionnaire.	After SFL, the proportion of bar workers reporting at least one respiratory symptom declined significantly by 18.1% for smokers and 25.1% for non-smokers. The level of wheezing among non-smokers declined significantly by 26.9% but not among smokers (5.9%); this interaction pre/post legislation, occurred also in ‘cough first thing in the morning’ and ‘cough in day or night’. Smokers had greater odds of reporting ‘shortness of breath’ than non-smokers (Odds ratio (OR) 2.02). There was greater odds of reporting this symptom before the legislation (OR 2.62).	Low
**Durham, 2011. Switzerland.** Canton of Vaud [78]								
To assess the ban’s impact in non-smokers and smokers by environmental tobacco smoke (ETS) exposure symptoms	Partial *Ban*: *15* September 2009	*Pre-ban*: 30 April 2009 *Post-ban*: 26 Sept 2010	Pre-posttest non-experimental design	105 adult hospitality workers. Dropout rate of 37%. Age, mean(before-after): 37.4–41.3.	Cough, wheezing, chest oppression, shortness of breath the four weeks prior.	Self reported questionnaire.	Percentage of reported symptoms pre/post-ban(p-value): cough 14%/16% (p 0.5), wheezing 7.84%/7.84%(p 0.646), chest oppression 5.77%/1.92%(p 0.309), shortness of breath 9.8%/7.84% (p 0.5)	Low
**Eagan, 2006. Norway.** [76]								
To examine the prevalence of respiratory symptoms among employees in the Norwegian hospitality industry, before and after enacting a SFL	Comprehensive *Ban*: 1 June 2004	*Pre-ban*: May 2004 *Post-ban*: October 2004	Pre-posttest non-experimental design	878 employees of Norwegian hospitality industry	Morning cough, daytime cough, phlegm cough, dyspnoea, wheezing, any symptom.	Medical Research Council questionnaire. Phone interviews	The prevalence of symptoms declined after the ban; for morning cough from 20.6% to 16.2% (p<0.01); for daytime cough from 23.2% to 20.9%; for phlegm cough from 15.3% to 11.8% (p 0.05); for dyspnoea from 19.2% to 13.0% (p 0.01); and for wheezing from 9.0% to 7.8%. The largest decline was seen among workers who gave up smoking, and with a positive attitude towards the law.	Low
**Eisner, 1998. California.** San Francisco [39]								
To study the respiratory health of bartenders before and after legislative prohibition of smoking in all bars and taverns	Comprehensive *Ban*: 1 January 1998	*Pre-ban*: December 1997 *Post-ban*: February 1998	Pre-posttest non-experimental design	Bartenders at a random sample of bars and taverns in San Francisco. 53 of the daytime bartenders. Mean age 42.5. Female 28%. -white 38%.	Wheezing, dyspnoea, morning cough, cough the rest of day, phlegm production and recent upper respiratory tract infections.	IUATLD Bronchial Symptoms Questionnaire	Thirty-nine (74%) of the 53 bartenders reported respiratory symptoms at baseline, while only 17 (32%) were still symptomatic at follow-up. Of the 39 bartenders reporting baseline symptoms, 23 subjects (59%) no longer indicated any respiratory symptoms after prohibition of smoking (p 0.001).	Low
**Farrelly 2005, US.** New York State [41]								
To assess the impact of New York’s law on respiratory symptoms in the past four weeks on hospitality workers’	Comprehensive Ban: 24 July 2003	*Pre-ban*: June- July 2003 *Post-ban*: -Oct- Nov 2003 -Feb—March 2004 - July- July 2004	Pre-posttest non-experimental design	68 workers in restaurants, bars, and bowling facilities. 47% completed the interview at 12 month follow up.Age (years) 18–26: 37.5%; 27–35: 12.5%; 36–45: 25%; >46: 25%. Male: 29.2%	Self reported respiratory symptoms in the past four weeks (wheeze, shortness of breath, morning cough, cough during the remainder)	IUATLD Bronchial Symptoms Questionnaire	There was no change in the overall prevalence of upper respiratory symptoms (p 0.1). Before the law, approximately 46% of respondents experienced any respiratory symptoms. This dropped by 37% to 29% not significantly. The most common respiratory symptom experienced was coughing during the day or at night (29%). The symptom scale shows that participants reported experiencing an average of 1.1 respiratory symptoms at baseline. By the 12 month follow up, there was a marginally significant change in coughing in the morning—dropping by 62%, from 21% to 8%.	Moderate
**Fernández, 2009. Spain.** [77]								
To evaluated self-reported respiratory health in hospitality workers in five regions of Spain before and after SFL. As a control group (without SFL) they studied hospitality workers in Portugal and Andorra.	Partial *Ban*: 1 January 2006	*Pre-ban*: October 2005 *Post-ban*: December 2006	Pre-posttest quasi-experimental design	Hospitality workers nonsmokers.117 in Spain and 20 workers in Portugal and Andorra followed up 12 months. Median age: Spain 39.4; Portugal-Andorra 37.1. Women in Spain 39.3% and 70.0% in Portugal-Andorra.	Breathless while wheezing, woken up with chest tightness, shortness of breath at rest, woken up by attack of shortness of breath, cough in the morning, cough during the rest, phlegm, asthma attack	European Community Respiratory Health Study (ECRHS) questionnaire. Face-to-face interviews.	The baseline prevalence of each symptom individually did not significantly change after the ban in Spain regardless of the type of post-ban smoking regulation, except for cough and phlegm among workers in totally smoke-free venues (from 40.6% to15.6% considered together). No changes were observed in the control regions. The prevalence of any respiratory symptom before the law was 32.5% in Spain. After the law, among workers in completely smoke-free venues, declined significantly (-71.9% change), but not in workers in venues where smoking was allowed on part (-57.1% change)or all of the premises (-19.4% change). In Portugal and Andorra, a borderline-significant decrease was observed (-61.9% change).	Moderate
**Goodman, 2007. Ireland.** Dublin [43]								
To examine the impact of this legislation on respiratory health effects in bar workers in Dublin.	Comprehensive *Ban*: 29 March 2004	*Pre-ban*: October 2003—March 2004 *Post-ban*: Sept 2004 -March 2005.	Pre-posttest non-experimental design	Bar staff volunteers (n 81), from pubs mostly different. Mean age of 47.9 at the preban assessment.	Respiratory symptoms.	Self reported IUATLD Questionnaire. California Environmental Protection Agency questionnaire	Significant improvements in cough in the morning, cough during the rest of day and phlegm production in nonsmokers (change -48%; -39%, and -41%, respectively). Decrease in any respiratory symptom from 86 to 61%(p<0.01).	Low
**Hahn, 2006. US.** Lexington [44]								
To evaluate the association between secondhand smoke (SHS) exposure and respiratory symptoms before and after a SFL on bar and restaurant workers	Comprehensive *Ban*: 27 April 2004	*Pre-ban*: before the law *Post-ban*: 3 and 6 months after	Pre-posttest non-experimental design	105 adult restaurant or bar workers, at 3 months postlaw, 71 and at 6 months 60. Female 63%, largelly white, mean age 26	Wheezing, dyspnoea, morning cough, cough during the rest, and phlegm production.	IUATLD Bronchial Symptoms Questionnaire	Significant decrease in symptom prevalence over time for most respiratory symptoms. In particular, for smokers and nonsmokers combined, morning cough, cough at other times during the day, all demonstrated a significant decline in prevalence between the prelaw period and the first postlaw interview, and this decline was maintained at 6 months postlaw.	Low
**Ho, 2010. China.** Hong Kong [47]								
To evaluated the effects of a SFL on the exposure of children to SHS at home and outside home in Hong Kong.	Partial *Ban*: January 2007	*Pre-ban*: January -March 2006 *Post- ban*: January—March 2008	Pre-posttest non-experimental design	3243 and 4965 primary 2–4 students in 2006 and in 2008 from 19–24 randomized schools, respectively, smokers excluded. The 2006 survey has 50.3% boys and a mean age of 8.3; the 2008 survey had 52.2% boys and a mean age of 8.6 years.	Frequent cough or phlegm were classified as having respiratory symptoms inside and outside home.	Self-administered questionnaire	The prevalence of respiratory symptoms increased slightly from 36.6% in 2006 to 38.4% in 2008 (p<0.001) for follow-up schools. Exposure SHS at home for 4–7 days per week was significantly associated with respiratory symptoms with an adjusted OR of 1.19 in 2008, compared with 1.09 in 2006. The each-day increase in home exposure was associated with 7% excess risk of respiratory symptoms in 2008 but no increased risk in 2006. Association with SHS outside home: for 4–7 days and respiratory symptoms seemed to be weaker in 2008 with OR of 1.54 compared with that of 2.06 in 2006, difference was insignificant. The each-day increase outside home decreased from 35% in 2006 to 20% in 2008 for the respiratory symptoms.	High
**Kim, 2015. Republic of Korea**. Seoul [50]								
To determine the effects of Korean smoking ban in restaurants and pubs in terms of air quality, biomarker levels, and health effects on staff.	Partial *Ban*: July 2013	*Pre-ban*: 29 April -June 2013 *Post-ban*: August-Sept 2013	Pre-posttest non-experimental design	95 staff members of restaurants or pubs who had never smoked or ex-smokers. 69% female. 19% ex-smokers. Mean age 47.4	Wheezing/whistling,shortness of breath, morning cough, rest of day or night cough, phlegm production	Self reported questionnaires based on IUATLD questionnaire.	The respiratory symptoms did not significantly differ among staff in any facilities before and after the ban. In < 150 m2 facilities (N = 45), 40% had respiratory symptoms before the law and 26% after the law (p 0.15). In ≥ 150 m2 facilities (N = 50), 36% had respiratory symptoms before the law and 26% after the law (p 0.22).	High
**Larsson, 2008. Sweden:** Stockholm, Göteborg, Malmö, Uppsala, Västerås, Linköping, Örebro, Östersund, Skövde [52]								
To evaluate the influence of the SFL among hospitality workers by examining the change in the rate of respiratory symptoms before and 12 months after enacting	Partial *Ban*: 1 June 2005	*Pre-ban*: April-May 2005 *Post-ban*: April-May 2006	Pre-posttest non-experimental design	91 hospitality workers of bingo halls and casinos. Altogether 71 of 91 (14 daily smokers and 57 nonsmokers) at follow-up: 70% female, 26% smokers	Respiratory symptoms reported	Self reported IUATLD Bronchial Symptoms Questionnaire.	In the 91 workers, all of the reported symptoms declined, and was significant for cough in the morning, cough during the rest of the day (not in nonsmokers). Among the smokers, there was no association between symptoms and period, but there only 14. Among the gaming workers, the OR declined more for cough in the morning (OR 0.23), cough the rest of the day (OR 0.10), and bringing up phlegm (OR 0.19, 0.02 to 1.61).	Moderate
**Li, 2013. China.** Shangai[74]								
To evaluate impact of SFL on respiratory symptoms among employees in five kinds of workplaces	Partial *Ban*: March 2010	*Pre-ban*: August 2009 *Post-ban*: September 2010	Pre-posttest non-experimental design	Employees of schools, kindergartens, hospitals, hotels, and shopping malls. At baseline, 2,254. Females 65.6%. Age mode: 30–49	Wheezing, dyspnea, morning cough, cough during the rest, phlegm production	IUATLD Bronchial Symptoms Questionnaire. Face-to-face interviews	The prevalence of respiratory and sensory symptoms among employees decreased significantly from 83% to 67%. There were statistically significant differences by type of establishment: shopping malls and schools had a sharp decrease in respiratory and sensory symptoms (p<0.05).	Moderate
**MacCalman, 2012. Scotland and England** [55]								
To investigate whether changes in self-reported symptoms and attitudes were related to participants' initial attitude towards SFL and to determine the nature of the relationship	Comprehensive *Ban*: *-*Scotland: March 2006 -England: July 2007	*Pre-ban* *Post-ban*: -2 month after -One year after	Pre-posttest non-experimental design, comparation between 2 studies.	548 bar workers, at follow-up 253. Age 31.3 (18.4, 66.7). Male 49.8%, 41.1% regular smoking.	Shortness of breath, tight chest, wheezing, phlegm production, morning cough and other cough, sore or dry throat	Self reported IUATLD Bronchial Symptoms Questionnaire.	The proportion of people reporting any symptoms was significantly reduced in both England (76% vs. 49%) and Scotland (67% vs. 87%). The proportion of bar workers in Scotland reporting wheezing reduced from 33 to 22%, while the reduction was from 35 to 10% of bar workers in England. The initial attitude to SFL did not have any effect on the change in respiratory symptoms reported by those in England (p 0.755);it did seem to have an effect in Scotland (p 0.042).	High
**Madureira, 2012. Portugal.** Vila Nova de Gaia. *The same data for Madureira*, *2014* [56] [57]								
To assess the impact of ETS exposure on respiratory symptoms among portuguese restaurant workers before and after a SFL	Partial *Ban*: 1 January 2008	*Pre-ban*: Oct-Dec 2006. *Post-ban*: 2010	Pre-posttest non-experimental design	52 restaurant workers. At 2 years of follow-up 47%. Mean age 30.8, 71% men, 46% smokers	Sore or dry throat, cough, tight chest, breathing difficulties such as breathe shortness or wheeze	Questionnaire, self reported symptoms	While 67% of workers reported at least one respiratory and sensory symptom in 2006 (pre-ban phase), only 29% noted the same symptoms in the postban phase. Similarly, self-reported respiratory symptoms decreased markedly 52% from the pre-ban to the post-ban phase	Moderate
**Menzies, 2006. Scotland**. Dundee and Perth [58]								
To investigate the association of SFL with symptoms and pulmonary function on bar workers nonsmokers and ex-smokers	Comprehensive *Ban*: 26 March 2006	*Pre-ban*: February 2006 *Post-ban*: May-June 2006	Pre-posttest non-experimental design	105 bar workers, without respiratory disease except asthma or rinitis (n = 23), 77 at follow-up. Mean age: 37.5, male 41%.	Wheeze, shortness of breath, cough, and phlegm	Abbreviated IUATLD Bronchial Symptoms Questionnaire	The percentage of bar workers with respiratory and sensory symptoms decreased from 79.2% before the SFL to 53.2% (p 0.001) and 46.8% (p<0.001) 1 and 2 months afterward. Significant improvements in the percentage of bar workers experiencing respiratory (total reduction, −20.8%, −7.6% to −33.9%; p 0.005) symptoms at 1 month after the ban and at 2 months (−35.1%, −22.2% to −47.9%; p<0.001).	Moderate
**Pearson, 2009. US.** Washington [62]								
To test the hypothesis that implementation of the SFL significantly reduces >50% respiratory and sensory symptoms reports	Comprehensive *Ban*: 2 January 2007	*Pre-ban*: December 2006 *Post-ban*: Febr 2007	Pre-posttest non-experimental design	52 bar employees nonsmoker from 41 randomly selected bars, post-ban assessment in 46. Male 89%	Shortness of breath, wheezing, coughing, and phlegm in the past four weeks.	IUATLD Bronchial Symptoms Questionnaire	The difference in respiratory symptom reports was inconclusive.	High
**Rajkumar, 2014. Switzerland.** Zurich, Basel City, and Basel County [63]								
To relate workplace SHS exposure in nonsmoking hospitality workers before and 6 to 12 months after a smoking ban to their respiratory health	Partial *Ban*: May 2010	*Pre-ban*: March 2010 *Post-ban*: 6 and 12 month after (until Dec 2010)	Pre-posttest non- experimental design	92 nonsmoking hospitality workers. Info baseline: 62% female, mean age 40.3, self-reported asthma 14.1%, allergic 65.2%.	Asthmatic symptoms (breathlessness, wheezing,chest tightness), chronic bronchitis (cough, phlegm), rhinitis (sneezing,running nose)	Computer-based interview adapted from a standardized questionnaire	Respiratory symptoms: Bronchitis symptoms (Cough 29.4%,Phlegm 12.0%); chronic bronchitis 2.2%; asthma symptoms 26.1%; allergy symptoms: rhinitis 22.8%. At baseline,the exposure-response model yielded an OR of 1.25(1.03 to 1.53) per cigarette/day increase in SHS exposure for cough and 1.13 (0.99 to 1.28) for chronic bronchitis. After SFL, the adjusted OR for cough was 0.59 (0.36 to 0.93) and 0.75 (0.55 to 1.02) for chronic bronchitis compared with the preban period.	Low
**Reijula, 2012. Finland** [65]								
To assess the impact of tobacco legislation in bars and restaurants.	Partial *Ban*: June 2007	*Pre-ban*: *2007* *Post-ban*: June 2009	Pre-posttest non-experimental design	1,008 restaurant workers in 2007 and 805 in 2009. 84.5% women. In 2007, 31% of women and 39% of men smokers. In 2009, 31% and 30%, respectively.	“has tobacco smoke in your workplace caused you respiratory or eye symptoms?’	Questionnaire surveys, paper form mailed to the participants (self-reported)	The prevalence of respiratory symptoms decreased from 18% to 4% (p < 0.0001) and was highest among bartenders of whom 32% reported respiratory symptoms in 2007 and 6% in 2009 (p < 0.0001). In 2009, the prevalence of respiratory symptoms among those who reported no exposure to ETS at work was 2% while among those who reported exposure to ETS for more than 4 hr a day was 21%.	Low
**Schoj, 2010. Argentina**. Neuquén [67]								
To evaluate the impact of SFL on respiratory symptoms and respiratory function among bar and restaurant workers	Comprehensive *Ban*: 15 November 2007	*Pre-ban*: October 2007 *Post-ban*: March 2008	Pre-posttest non-experimental design	134 non-smokers bar and restaurant workers. 80 at follow up: 38.7% women and 6.2% had a history of asthma. Mean age 34.3.	Cough, phlegm production, wheezing and dyspnoea. History of asthma	IUATLD Bronchial Symptoms Questionnaire	An important reduction in respiratory symptoms (from a pre-ban level of 57.5% to a post-ban level of only 28.8%). Respiratory symptoms that declined after prohibition: such as cough, cough at night, dyspnoea on exertion and at rest, and tightness in the chest.	Low
**Wilson, 2012. US.** Michigan [73]								
To determine the impact on bar employee’s health and exposure to SHS before and after the implementation of a SFL	Comprehensive *Ban*: 1 May 2010	*Pre-ban*: 6 weeks before *Post-ban*: 6–10 weeks after	Pre-posttest non-experimental design	40 bar employees ≥ 18 years; never smoked or ex-smokers, lived in a smoke-free household. 70% women, mean age 44.8. 95% whites.	Morning cough, daytime cough, phlegm, shortness of breath, wheezing and allergic symptoms.	A self-administered respiratory questionnaire by the University of Minnesota, Masonic Cancer Center.	There was a significant improvement in all six self-reported respiratory symptoms (p<0.001). Respiratory symptom (p pre-law/post-law): Allergic symptoms (p<0.001), Wheezing (p 0.050), Shortness of breath (p 0.048), Phlegm production (p 0.021), Daytime cough (p .018), Morning cough (p 0.003)	Moderate
**Sensory symptoms**								
**Allwright, 2005.The Republic and Northern Ireland** (three areas in the Republic-Dublin, Cork, and County Galway- intervention- and one area in Northern Ireland -control) [31]								
To compare respiratory health in bar staff in the Republic of Ireland before and after the law and to compare these changes with changes observed in Northern Ireland.	Comprehensive. *Ban*: March 2004	*Pre-ban*: September 2003 *Post-ban*: March 2005	Pre-posttest quasi-experimental design	226 participants at baseline, 213 in the follow-up. Rep Ireland: Mean age 45.5, 17% women. North Ireland: Mean age 36.1, 25% women.	Red eyes, sore throat	IUATLD Bronchial Symptoms Questionnaire.	After the ban, reporting any sensory symptom dropped from 67% to 45% (p < 0.001), reflecting significant declines in reporting red eyes (p < 0.001) and sore throat (p 0.004). In Northern Ireland, the proportion reporting any sensory symptom declined from 75% to 55% (p 0.13). The adjusted RR for the number of sensory symptoms dropped in both regions (by 50% in the Republic and by 44% in Northern Ireland).	Low
**Ayres, 2009. Scotland** [32]								
To examine changes in prevalence of self-reported respiratory and sensory symptoms of bar workers after SFL was introduced	Comprehensive *Ban*: 26 March 2006	*Pre-ban*: 7 Jan 2006 *Post-ban*: -May- July 2006 - Jan -March 2007	Pre-posttest non-experimental design	371 bar workers, including managers, owners and bar staff non smokers, smokers and ex-smokers. Only 177 at 3 phases. Age 29.5, male 51%	Self-reported eyes, nose, throat, any sensory symptom	IUATLD Bronchial Symptoms Questionnaire	Of the 191 (51%) workers seen at 1-year follow- up, the percentage reporting any sensory symptoms fell from 75% to 64% (p 0.02), effects being greater at 2 months, probably partly due to seasonal effect. Reduction in ‘‘any” sensory symptom: 69% falling to 54%, p 0.011. For non-smokers (n = 57) the reductions in reported symptoms were significant for red/irritated eyes (44% to 18%).	Low
**Bannon 2009, North of Ireland.** Belfast [40]								
To assess, before and after the introduction of the SFL, bar workers’ self-reported levels of sensory symptoms	Comprehensive *Ban*: April 2007	*Pre-ban*: March 2007 *Post-ban*: July 2007	Pre-posttest non-experimental design	97 (pre-ban) and101 (post- ban) bar workers of the 35 Belfast bars. Female before 39, after 42; Male before 58, after 59. The majority rank of age: 16–25 and 26–35 years.	Red or irritated eyes, runny nose, sneezing or nose irritation, sore or scratchy throat, at least one sensory symptom	A short self-completing questionnaire similar to IUATLD Questionnaire.	Reductions for sensory symptoms ranged from 7.3% -17.7% for smokers, from 29.6%- 46.8% for non- smokers.The proportion of bar workers reporting ‘at least one sensory symptom’ declined by 36% (p 0.001) for non-smokers, not significantly for smokers. Running nose: adjusted OR 2.38, p 0.005. ‘Sore or scratching throat’; its level declined among non-smokers by 41.5% (p< 0.001),not significant for smokers.Eye irritation for non-smokers:OR 7.72 (p < 0.001);for smokers,OR 2.28, p 0.08.	Low
**Durham, 2011. Switzerland.** Canton of Vaud [78]								
To assess the ban’s impact in non-smokers as well as smokers on ETS exposure symptoms	Partial *Ban*: *15* September 2009	*Pre-ban*: 30 April 2009 *Post-ban*: 26 Sept 2010	Pre-posttest non-experimental design	105 hospitality workers, 66 after one year. Mean age (before): 37.4. Smoking status (before-after): 61%-54.6%.	Red eyes,irritated eyes, irritated nose, runny nose, sneezing	Self reported questionnaire.	Typical ETS exposure symptoms in the four weeks prior were generally reduced at follow-up. Red and irritated eye symptoms decreased from 26.79% and 31.48% to 12.5% and 11.11% respectively (p 0.047 and 0.009), sneezing also decreased significantly from 23.53% prior to the ban to 7.84% after- wards	Low
**Eisner, 1998. California.** San Francisco [39]								
To study the respiratory health of bartenders before and after SFL	Comprehensive *Ban*: 1 January 1998	*Pre-ban*: Dec 1997 *Post-ban*: Feb 1998.	Pre-posttest non-experimental design	53 of the daytime bartenders. Mean age 42.5. Female 28%. Nonwhite 38%.	Red or irritated eyes; runny/irritation nose, sneezing; scratchy throat	IUATLD Bronchial Symptoms Questionnaire.	51 bartenders (77%) initially reported sensory irritation symptoms. At follow-up, 32 (78%) had resolution of symptoms (p<0.001). After excluding the 8 subjects who reported a recent upper respiratory infection, at follow-up interview, 79% no longer reported any sensory symptoms (p<0.001).	Low
**Farrelly 2005, US.** New York State [41]								
To assess the impact of New York’s law on sensory symptoms in the past four weeks on hospitality workers’	Comprehensive *Ban*: 24 July 2003	*Pre-ban*: June-July 2003 *Post-ban*: -15 Oct-19 Nov 2003 - 20 Feb -23 March 2004 - July-July 2004	Pre-posttest non-experimental design	68 workers in restaurants, bars, and bowling facilities. 69%, 56% and 47% completed both the interview at the three, six, and 12 month follow up studies, respectively. Age (years) 18–26: 37.5%; 27–35: 12.5%; 36–45: 25%; >46: 25%. Male: 29.2%	Self reported sensory irritation in the past four weeks (eye, nose, throat)	IUATLD Bronchial Symptoms Questionnaire	At baseline, 88%(95% CI 66% to 96%) of respondents experienced any one of three sensory symptoms and reported an average of 1.6 sensory symptoms. By the 12 month follow up, the presence of one or more sensory symptoms decreased by 57% (p 0.01), from 88% to 38%(20% to 59%) (p 0.01), and all individual symptoms declined significantly. Similarly, the total number of sensory symptoms experienced (symptom scale) declined by 69% (p 0.01) from baseline (1.6) to the 12 month follow up (0.5)	Moderate
**Goodman, 2007. Ireland.** Dublin [43]								
To examine the impact of this legislation on respiratory health effects in bar workers in Dublin.	Comprehensive *Ban*: 29 March 2004	*Pre-ban*: Oct 2003 -March 2004 *Post-ban*: Sept 2004 -March 2005.	Pre-posttest non-experimental design	Bar staff volunteers (n 81), from pubs mostly different. Mean age 47.9 years at the preban assessment.	Sensory symptoms.	IUATLD Bronchial Symptoms Questionnaire.California Environmental Protection Agency questionnaire	The results showed significant improvements in sensory irritant symptoms in smokers and non-smokers, but smokers benefited less.	Low
**Hahn, 2006. US.** Lexington [44]								
To evaluate the association between SHS exposure and sensory symptoms before and after the SFL	Comprehensive *Ban*: 27 April 2004	*Pre-ban*: before the law *Post-ban*: 3 and 6 months after	Pre-posttest non-experimental design	105 adult restaurant or bar workers, at 3 months postlaw, 71 and at 6 months 60. Female 63%, largelly white, Mean age 26	Red, irritated eyes; runny nose, sneezing, or irritated nose; and scratchy or sore throat	Self reported IUATLD Bronchial Symptoms Questionnaire.	Prevalence of sensory symptoms and comparisons by time, smoking status, for smokers and nonsmokers combined red eyes, and runny nose demonstrated a significant decline in prevalence between the prelaw period and the first postlaw interview, and this decline was maintained at 6 months postlaw.	Low
**Kim, 2015. Republic of Korea**. Seoul [50]								
To determine the effects of Korean smoking ban in restaurants and pubs in terms of air quality, biomarker levels, and health effects on staff.	Partial *Ban*: July 2013	*Pre-ban*: April -June 2013 *Post-ban*: August–Sept 2013	Pre-posttest non-experimental design	95 staff members of restaurants or pubs who had never smoked or ex-smokers. 69% female. 19% ex-smokers. Mean age 47.4	Red or irritated eye, runny or sneezing nose, sore or scratchy throat	Self reported questionnaires that were based UATLD Questionnaire.	The self-reported health effects on sensory symptoms were estimated by area regardless of the type of facility due to the low incidence of symptoms in each type of facility. The sensory symptoms among the staff in ≥150 m^2^ facilities (n = 50) significantly decreased from 52% at baseline to 40% after the ban, whereas the staff from <150 m^2^ facilities (n = 45) did not exhibit a significant change in symptoms	High
**Larsson, 2008. Sweden:** Stockholm, Göteborg, Malmö, Uppsala, Västerås, Linköping, Örebro, Östersund, Skövde [52]								
To evaluate the influence of the SFL among hospitality workers by examining the change in the rate of sensory symptoms before and after	Partial *Ban*: 1 June 2005	*Pre-ban*: April-May 2005 *Post-ban*: April-May 2006	Pre-posttest non-experimental design	91 hospitality workers of bingo halls and casinos. 71 (14 daily smokers and 57 nonsmokers) at follow-up: 70% female, 26% smokers	Sensory symptoms	Self reported IUATLD Bronchial Symptoms Questionnaire.	In the entire study population, all of the reported symptoms declined, and the decline was statistically significant for questions about eye irritation, nose irritation, and throat symptoms. Among the smokers, there was no notable association between symptoms and period. The sensory symptoms declined somewhat more among no gaming workers.	Moderate
**Li, 2013. China.** Shangai [74]								
To evaluate the compliance with the SFL as well as its impact on sensory symptoms among employees in five kinds of workplaces	Partial *Ban*: March 2010	*Pre-ban*: August 2009 *Post-ban*: September 2010	Pre-posttest non-experimental design	Employees of schools, kindergartens, hospitals, hotels, and shopping malls. At baseline, 2,254. At follow-up 1832. Females 65.6%. Mode age: 30–49	Red or irritated eyes, runny, sneezing nose, and sore or scratchy throat.	IUATLD Bronchial Symptoms Questionnaire. Face-to-face interviews	The prevalence of respiratory and sensory symptoms among employees decreased from 83% to 67%. There were statistically significant differences by type of establishment: shopping malls and schools had a sharp decrease in respiratory and sensory symptoms. There was no significant change in kindergartens after the legislation.	Moderate
**MacCalman, 2012. Scotland and England** [55]								
To investigate whether changes in self-reported symptoms and attitudes were related to participants' initial attitude towards SFL	Comprehensive *Ban*: *-*Scotland: March 2006 -England: July 2007	*Pre-ban* *Post-ban*: -2 month after -One year after	Pre-posttest non-experimental design, comparation between 2 studies.	548 bar workers, at follow-up 253. Age 31.3 (18.4, 66.7). Male 49.8%, 41.1% regular smoking.	Runny nose, dry, itching, irritated, wathery eyes, and sore scratchy throat	Self reported IUATLD Bronchial Symptoms Questionnaire.	Initial attitude did not have an effect on the change in symptoms reported by those in England. The proportion of people reporting any symptoms was significantly reduced in both England and Scotland. The proportion of people reporting any symptoms was significantly reduced from pre-ban period to one year after, in both England (76% vs. 49%) and Scotland (67% vs. 87%). The initial attitude to SFL did not have any effect on the change in symptoms reported by those in England.	High
**Madureira, 2012. Portugal.** Vila Nova de Gaia. *The same data for Madureira*, *2014* [56] [57]								
To assess the impact of ETS exposure on respiratory symptoms among portuguese restaurant workers before and after a SFL	Partial *Ban*: 1 January 2008	*Pre-ban*: Oct-Dec 2006. *Post-ban*: 2010	Pre-posttest non-experimental design	52 restaurant workers. At 2 years of follow-up 47%. Mean age 30.8, 71% men, 46% smokers.	Mucosal irritation or dry, itching, irritated, or watery eyes; nasal problems	Self reported questionnaire.	The most common indoor air-related symptoms reported by the participants in the pre-ban phase were dry, itching, irritated, or watery eyes (48%). In the postban phase the most common symptoms were dry, irritated, or watery eyes, fatigue, and headache (21%). Reported at least one respiratory and sensory symptom felled from 67% to 29%.	Moderate
**Menzies, 2006. Scotland**. Dundee and Perth [58]								
To investigate the association of SFL with symptoms on bar workers nonsmokers and ex-smokers	Comprehensive *Ban*: 26 March 2006	*Pre-ban*: Feb 2006 *Post-ban*: May-June 2006	Pre-posttest non-experimental design	105 bar workers, without respiratory disease except asthma or rinitis (n = 23), 77 completed the study. Mean age: 37.5, male 41%,	Red or irritated eyes, painful throat and nasal itch, runny nose, and sneeze	Abbreviated IUATLD Bronchial Symptoms Questionnaire	The percentage of bar workers with respiratory and sensory symptoms decreased from 79.2% (n = 61) before the smoke-free policy to 53.2% (p 0.001) and 46.8% (p<0.001) 1 and 2 months afterward. Significant improvements in the percentage of bar workers experiencing sensory (total reduction, −31.2%; −18.1% to −44.3%; p<0.001) symptoms at 1 month after the ban and at 2 months (−35.1%, −21.7% to −48.4%).	Moderate
**Pearson, 2009. US.** Washington [62]								
To test the hypothesis that implementation of the SFL reduces >50% respiratory and sensory symptoms reports	Comprehensive *Ban*: 2 January 2007	*Pre-ban*: Dec 2006 *Post-ban*: Feb 2007	Pre-posttest non-experimental design	52 elegible bar employees nonsmoker (94% follow-up), final sample size of 46. Male 89%	Eye, nose, and throat irritation in the past four weeks	IUATLD Bronchial Symptoms Questionnaire	Sensory symptoms reports declined significantly by 70% to 100%, from a median of 2 to a median of 0.	High
**Reijula, 2012. Finland** [65]								
To assess the impact of tobacco legislation in bars and restaurants.	Partial *Ban*: June 2007	*Pre-ban*: 2007 *Post-ban*: June 2009	Pre-posttest non-experimental design	1008 restaurant workers in 2007. 84.5% women. Smokers In 2007: 31% of women, 39% of men.	‘has tobacco smoke in your workplace caused your eye symptoms?”	Questionnaire surveys, paper form mailed (self-reported)	The prevalence of eye symptoms decreased from 23% to 6% (p < 0.0001). The prevalences of eye symptoms among those who reported no exposure to ETS at work were 2% and among those who reported exposure to ETS for more than 4 hr a day 39%.	Low
**Schoj, 2010. Argentina**. Neuquén [67]								
To evaluate the impact of SFL on sensory irritation symptoms among bar and restaurant workers	Comprehensive *Ban*: 15 November 2007	*Pre-ban*: Oct 2007 *Post-ban*: March 2008	Pre-posttest non-experimental design	134 non-smokers bar and restaurant workers. 80 at follow up: 38.7% women Mean age 34.3	Red or irritated eyes; sore and scratchy throat, sneezing and running nose.	IUATLD Bronchial Symptoms Questionnaire	The reduction of sensory irritation symptoms was even higher than the reduction of respiratory symptoms. From 86.3% of workers who reported at least one sensory irritation symptom in October 2007, only 37.5% reported the same symptoms in March 2008.	Low
**Wieslander, 2000. Sweden** [72]								
To determine the influence of a ban on smoking on commercial airlines on nonspecific symptomatology	Labor: smoking ban on intercontinental flights *Ban*: September 1997	*Pre-ban*: end of august 1997 *Post-ban*: 2 weeks after, Sept 1997	Pre-posttest non-experimental design	Non asthmatic commercial aircrews. In pre-ban flight n = 39: 35% women, 8% asthmatic, 27% smokers. Post-ban flight n = 41: 0% asthma, 21% smokers	Symptoms (nasal, ocular, dermal and general symptoms).	Medical self-reported questionnaire, medical examination	There were fewer ocular symptoms. A numerical decrease of all types of individual symptoms occurred for the nonasthmatic subjects after the smoking ban. The occurrence of more than 1 ocular symptom was decreased from 55% to 11% (P = 0.004) after the smoking ban. The total symptom score was higher for the smoke conditions (mean 3.9) than for the nonsmoke conditions (mean 1.4) (p 0.05).	High
**Spirometry**								
**Durham, 2011. Switzerland.** Canton of Vaud [78]								
To assess the ban’s impact in non-smokers as well as smokers on lung functions, ETS exposure symptoms and the perceived impact of the law.	Partial *Ban*: *15* September 2009	*Pre-ban*: 30 April 2009 *Post-ban*: 26 September 2010	Pre-posttest non-experimental design	105 adult hospitality workers, 66 after one year. Age, mean(before): 37.4. Smoking status(before-after): 61%-54.6%.	Lung function by spirometry: forced expiratory volume in one second (FEV1) and forced vital capacity (FVC)	Spirometry with an EasyOne portable spirometer.	Both baseline FEV1 and FVC are reduced to 90% of the predicted value compared to never smoking adults. FEV1 values were lower in men (87.84%) than in women (91.76%) and in smokers (88.68%) than in non-smokers (91.58%), similar values for FVC. At one-year there was a significant increase in FVC from 90.42% to 93.05%, marked in women (+3.07%), non-smokers (+3.91%) and older participants (+4.22%). Asthmatic participants had an almost significant increase in FEV1.	Low
**Eisner, 1998. California.** San Francisco [39]								
To study the respiratory health of bartenders before and after legislative prohibition of smoking in all bars and tavern	Comprehensive *Ban*: January 1, 1998	*Pre-ban*: December 1997 *Post-ban*: Feb 1998.	Pre-posttest non-experimental design	54 of the daytime bartenders (81%) completed spirometry; 98% at follow-up. Mean age 42.5. Female 28%. Nonwhite 38%.	FEV1 and FVC	Spirometry	After prohibition of smoking, the mean FVC and FEV1 both increased at follow-up. Flow rate at midlung volumes (FEF25%-75%), which was highly variable, declined during the study period	Low
**Goodman, 2007. Ireland.** Dublin [43]								
To examine the impact of this legislation on respiratory health effects in bar workers in Dublin.	Comprehensive *Ban*: 29 March 2004	*Pre-ban*: Oct 2003—March 2004 *Post-ban*: Sept 2004 -March 2005.	Pre-posttest non-experimental design	Bar staff volunteers (n 81), from pubs mostly different. Mean age of 47.9 years at the preban assessment.	FEV1, FVC, forced expiratory flow of 25 to 75% (FEF25–75), peak expiratory flow (PEF), and others parameters	Spirometry	FVC increased significantly in never-smokers and ex-smokers, whereas it declined in current smokers. Although FEV1 did not change significantly in any group, it tended to increase in nonsmokers. The TLC increased in never-smokers and ex-smokers but not in smokers. PEF increased significantly in never-smokers, and it tended to decline in current smokers. FEF25–75 decreased in never-smokers and ex-smokers.	Low
**Larsson, 2008. Sweden:** Stockholm, Göteborg, Malmö, Uppsala, Västerås, Linköping, Örebro, Östersund, Skövde [52]								
To evaluate the influence of the SFL among hospitality workers by examining the change in the rate of lung function before and 12 months after enacting	Partial *Ban*: 1 June 2005	*Pre-ban*: April-May 2005 *Post-ban*: April-May 2006	Pre-posttest non-experimental design	91 hospitality workers of bingo halls and casinos. 71 (14 daily smokers and 57 nonsmokers) at follow-up 12 months after the ban: 70% female, 26% smokers	FEV1 and FVC	Spirometry	The mean FEV1 for the nonsmokers was defined as 100% at the baseline, after 12 months, it had declined to 99%. Among the smokers, it fell from 93% to 90%. The mean FVC of the nonsmokers was 93% at the baseline and 92% after 12 months, and, among the smokers, it fell from 94% to 92%. The regression analysis produced adjusted coefficients of 0.020 (p 0.566) for the nonsmokers and -0.004 (p 0.957) for the smokers.	Moderate
**Menzies, 2006. Scotland**. Dundee and Perth [58]								
To investigate the association of SFL with symptoms and pulmonary function on bar workers	Comprehensive *Ban*: March 26, 2006	*Pre-ban*: February 2006 *Post-ban*: 1 and 2 months after (May-June)	Pre-posttest non-experimental design	105 bar workers nonsmokers and ex-smokers, without respiratory disease except asthma or rinitis (n = 23), 77 completed the study. Mean age: 37.5, male 41%	FEV1, PC10, forced exhaled nitric oxide (FENO)	Spirometry with a portable handheld spirometer	FEV1 increased from 96.6% predicted to 104.8% (change, 8.2%, 3.9% to 12.4%; p<0.001) and then 101.7% (change, 5.1%, 2.1% to 8.0%; p 0.002). The greatest gains were in the asthmatic cohort	Moderate
**Schoj, 2010. Argentina**. Neuquén [67]								
To evaluate the impact of SFL on respiratory function among bar and restaurant workers	Comprehensive *Ban*: 15 November 2007	*Pre-ban*: Oct 2007 *Post-ban*: March 2008	Pre-posttest non-experimental design	134 non-smokers workers. 80 at follow up: 38.7% women. 6.2% had a history of asthma. Mean age 34.3	FEV1 and FVC	Spirometric measurements with a portable spirometer.	They found a significant improvement in FVC (mean 96, SD 12 to 88). They did not find any significant differences in FEV1 measurements (mean 90, SD 17 to 70).	Low
**Skogstad, 2006. Norway**. Oslo [68]								
To compare cross shift changes in pulmonary function among employees in restaurants and bars before and after enforcement of SFL	Comprehensive *Ban*: 1 June 2004	*Pre-ban*: May 2004 *Post-ban*: February 2005	Pre-posttest non-experimental design	93 subjects employed. At follow up 69 individuals: 35 women, 26 non-smokers and 11 asthmatics.	FVC, FEV1 and FEF 25–75	Spirometry	The cross shift reduction in FVC not significant changed following the SFL. The reduction FEV1 during a workshift, almost significantly reduced. The reduction in FEF25–75% changed significantly from 199 ml/s to 64 ml/s. Among non-smokers and asthmatics, the reduction in FEV1 and FEF25–75 was significantly larger before compared to after. The mean pre-ban cross shift fall in FEV1 was 120 ml compared to 37ml (p 0.03) post-ban and FEF25–75 decreased from 218 ml/s to 65 ml/s (p 0.01).	Moderate
**Vinnikov, 2013. Kyrgyzstan.** Tyan Shan mountain [71]								
To assess whether annual lung function change associated with chronic intermittent hypoxia and smoking exposures differed after a workplace smoking ban	Labor *Ban*: January 2009	*Pre-ban*: 3 years before(2006–2009) *Post-ban*: 2 years until 2011	Pre-posttest non-experimental design	109 high-altitude gold-mine healthy miners at high altitude (4000 m), local subjects, 59% active mine production workers. Women, n = 13 (0 smokers). Males: 55% current smokers at the baseline. Mean age 32.5 years.	FEV1 and FVC, FVC% pred, and the FEV1/FVC ratio	MicroMedical MicroLab (UK) equipment, Smokerlyzer CO (Bedfont, UK).	There was a 115 ± 9 mL annual decline in lung function before the ban but a 178 ± 20 mL per annum increase in the final 2 years (P < 0.001). There was a 2.1 ± 0.3% annual decline in FEV1% pred until the ban and a 4.6 ± 0.5% increase after the ban. FVC, as with FEV1, the magnitude of the change was clinically relevant, lung function declined before the smoking ban and improved after it. For FVC and FVC% pred, annual change was statistically significant in both time periods; for FEV1/FVC, the decline before the ban was statistically significant, but the positive slope after the ban was not statistically significant.	Low
**Asthma symptoms**								
**Dove, 2010. US** [37]								
To investigate the association between smoke-free laws and asthma prevalence and severity among nonsmoking youth	Different types of laws and differents locations	From 1999 to 2006, in 2-year cycles	Posttest quasi-experimentaldesign.	Size of 8800, USA nonsmoking children and adolescents participants aged 3–15 years	Asthma prevalence. Asthmatic symptoms	National Health and Nutrition Examination Survey (NHANES). Self reported asthma severity.	SFL were associated with lower odds of asthmatic symptoms (OR: 0.67, 0.48 to 0.93) and trended toward lower odds o f ever having asthma with current symptoms (OR 0.74, 0.53 to 1.03) and asthma attacks (OR 0.66, 0.28 to 1.56)	High
**Asthma severity**								
**Kalkhoran, 2014. Uruguay.** Montevideo [79]								
To evaluate the impact of a SFL on non-hospital emergency care visits, hospitalizations for bronchospasm, and bronchodilator use	Comprehensive *Ban*: 1 March 2006	*Pre-ban*: 3 years before *Post-ban*: 5 years after. February 28, 2011	Non-experimental time series design	180,000 people in Montevideo aged ≥ 15 year with a non-hospital emergency visit for bronchospasm	Puffs of salbutamol, and ipratropium, administered per patient per month in the non- hospital emergency setting and during transfer to the hospital.	Electronic medical record of Servicio de Urgencia, Asistencia y Traslado. Medical Emergency Service.	Total monthly puffs of salbutamol and ipratropium administered in the non-hospital emergency setting decreased by 224 (-372 to –76) and 179 (–340 to –18.6), respectively, from means of 1,222 and 1,007 before the law	Low
**Menzies, 2006. Scotland**. Dundee and Perth [58]								
To investigate the association of SFL with symptoms and pulmonary function on bar workers nonsmokers and ex-smokers	Comprehensive *Ban*: 26 March 2006	*Pre-ban*: Feb 2006 *Post-ban*: May-June 2006	Pre-posttest non-experimental design	105 bar workers, without respiratory disease except asthma or rinitis (n = 23), 77 completed the study. Mean age: 37.5, male 41%	Asthma quality- of-life scores, data on current prescribed asthma medication	Self administration Juniper quality-of-life scores.	Asthmatic bar workers:Juniper quality-of-life scores increased from 80.2 to 87.5 points (7.3 points, 0.1 to 14.6 points; p 0.049). Indeed, a 0.5-point improvement is regarded as a clinically significant change in this tool.	Moderate
**Other**								
**Dove, 2010. US** [37]								
To investigate the association between smoke-free laws and persistent ear infection among nonsmoking youth (aged 3–15 years).	Different types of laws in differents settings	From 1999 to 2006, in 2-year cycles	Posttest quasi-experimental design	Size of 8800, USA nonsmoking children and adolescents participants aged 3–15 years	Persistent ear infection (3 or more ear infections in the previous year).	NHANES. Self reported persitent ear infection.	Youth living in smoke-free counties had approximately half the prevalence of persistent ear infections in the previous year (3.4%) compared with youth living in counties without a smoke-free law (6.1%). After adjustment for covariates, this difference no longer persisted. Youth living in the South and Midwest were more likely to have persistent ear infection and were less likely to live in a smoke-free county. The OR adjusted for all covariates except region was 0.64 (0.37 to 1.10)	High

CI: confidence interval. CO: carbon monoxide. ECRHS: European Community Respiratory Health Study. ETS: environmental tobacco smoke. FEF 25–75%: forced expiratory flow of 25 to 75%. FENO: forced exhaled nitric oxide. FEV1: forced expiratory volume in one second. FVC: forced vital capacity. ICD: International Classification of Disease. IQR: Interquartile range. IUATLD: International Union Against Tuberculosis and Lung Disease. NHANES: National Health and Nutrition Examination Survey. NS: no significant. OR: odds ratio. PC10: provocative concentration of methacholine causing a 10% decrease in FEV1. PEF: peak expiratory flow. RR: rate ratio. RV: residual volume. SD: standard deviation. SE: standard error. SFL: smokefree law. SHS: secondhand smoke. TLC: total lung capacity

**Table 2:** Summary of studies on the impact of smoke-free legislation on admissions.

**Asthma**								
**Aims**	**Legislation**	**Study period**	**Study design**	**Study participants and size**	**Variables**	**Source of information**	**Summary of findings**	**Risk of Bias**
**Croghan, 2015. US.** Minnesota [34]								
To evaluate the impact of a state- wide clean indoor air law on the frequency of emergency department (ED) visits for asthma.	Comprehensive *Ban*: 16 May 2007. Enacted on 1 October 2007	*Pre-ban*: 1 January 2005 *Post-ban*: 31 Dec 2009	Non-experimental time series design	2013 general population, 147,066 (86.5% white, 51.1% female), median age 37 for asthma; 47 for adults, 6 for children	ED visits for primary diagnosis of asthma (ICD,9th code 493) adjusted by age and sex	Medical records of Mayo Clinic and Olmsted Medical Center	5,906 ED visits with a primary diagnosis of asthma during the 5-year study period. A significant reduction was detected in asthma-related ED visits (RR 0.814) following the enactment of the SFL. The reduction was observed in both adults (RR 0.840) and children (RR 0.751).	Low
**Dove, 2010. US** [37]								
To investigate the association between smoke-free laws and asthma prevalence, and severity among nonsmoking youth	Different types of laws in different settings	From 1999 to 2006, in 2-year cycles	Posttest quasi-experimental design	Size of 8800, USA nonsmoking children and adolescents participants aged 3–15 years	Asthma severity (asthma attack or emergency-department visit for asthma)	NHANES. Self reported asthma severity.	Smoke-free laws were associated with lower odds of emergency-department visits for asthma (OR: 0.55, 0.27 to1.13), although these results were not statistically significant	High
**Gaudreau 2013. Canada.** Prince Edward Island (PEI) [42]								
To examine changes in hospital admission rates for respiratory (asthma) conditions were examined before and after a SFL.	Comprehensive *Ban*: 1 June 2003	*Pre-ban*: April 1995 -May 2003 *Post-ban*: June 2003 –Dec 2008	Quasi-experimental time series design	PEI population of 143,000. Province of New Brunswick (NB) population 729,995.	Asthma admissions were divided into pediatric admissions under 15 years of age and adult admissions over 15 years of age (ICD-9 493 and ICD-10 J45-J46)	PEI acute care hospitals registers. Census data from 2001 and 2006	Crude annual admissions for pediatric (p<0.01) and adult asthma (p<0.01) trended downward from 1995 to 2008. Change in monthly means of admission for respiratory and control conditions after the SFL by sex, per 100,000 population 1995 to 2008: pediatric asthma male 0.97(p 0.89), female 0.91(p 0.81); adult asthma male 1.42 (p 0.42), female 1.45 (p 0.17).	Low
**Head, 2012. US.** Beamont (Texas) [45]								
To examine hospital discharge data on 5 tobacco-related diagnoses before and after implementation of a smoking ban	Comprehensive *Ban*: July 2006	*Pre- Ban*: July 2004-June 2006. *Post Ban*: July 2006-June 2008	Pre-posttest quasi-experimental design	All residents. Intervention city: Beaumont (≈115,000). Control city: Tyler (≈ 87,600). Mean age: 33 years, Beaumont non-Hispanic black 50%	Hospital discharge rates for asthma (ICD-9 493)	The Texas Department of State Health Services Discharge. Census information	Discharge rates in the intervention city (Beaumont) declined significantly for asthma (RR 0.69; 0.52–0.91) for whites only. Discharge rates for asthma in the control city (Tyler) did not change.	High
**Herman, 2011. US.** Arizona [46]								
To examined the impact of a comprehensive statewide smoking ban on hospital admissions for asthma.	Comprehensive *Ban*: May 1, 2007	*Pre-ban*: Jan 2004- April 2007. *Post-ban*: May 2007 to May 2008.	Quasi-experimental time series design	All Arizona residents (general population).	Primary diagnoses for acute asthma admissions (ICD-9 code: 493).	Hospital admission data gathered by the 87 hospitals in Arizona	The estimated change in admissions for asthma in ban counties is negative and statistically significant. There is evidence that the following reductions (and percentage reductions) in hospital admission cases in the non previous ban counties from May 1, 2007, to May 31, 2008, are attributable to the statewide ban: 249 (22%) fewer asthma cases.	Moderate
**Humair, 2014. Switzerland.** Canton of Geneva [48]								
To evaluate the effect of the public smoking ban on hospital admissions for acute respiratory diseases	Comprehensive *Ban*: 1 July 2008	*Pre-ban*: July 2006-July 2008 *Post-ban*: 1rst ban: July 2008- Sept 2008 No ban: Oct 2008- Oct 2009 2nd Ban: Oct 2009-Dec 2010	Pre-posttest non-experimental design	Patients aged ≥ 16 admitted to University Hospitals of Geneva, with about 450,000 inhabitants. 5345 total admissions: 60% males, mean age of 67. 204 patients with a first hospitalization for acute asthma	First hospitalization for asthma (ICD-10 codes: J45-46).	Hospital database of the University Hospitals of Geneva. Census data of Geneva	Despite variations in the number of admissions, adjusted IRR of hospitalizations for acute asthma did not significantly change throughout the 4 periods (IRR final period 1.17; CI 0.82–1.66; p 0.81 for all patients and IRR final period 1.36; CI 0.91–2.01, p 0.42 for Geneva residents only)	Moderate
**Kalkhoran, 2014. Uruguay.** Montevideo [79]								
To evaluate the impact of Uruguay’s national 100% smokefree legislation on non-hospital emergency care visits, hospitalizations for bronchospasm, and bronchodilator use	Comprehensive *Ban*: March 1, 2006	*Pre-ban*: 3 years before *Post-ban*: 5 years after. February 28, 2011	Non-experimental time series design	180,000 people in Montevideo, Uruguay aged ≥ 15 year with a non-hospital emergency visit for bronchospasm	Number of monthly visits for bronchospasm from the non-hospital emergency service, number of individuals subsequently hospitalized (ICD-10 J45)	Electronic record of Servicio de Urgencia, Asistencia y Traslado.	The incidence of non-hospital emergency visits for bronchospasm decreased by 15% (IRR 0.85, 0.76 to 0.94) following implementation of the law. Hospitalizations for bronchospasm did not change significantly (IRR 0.89, 0.66 to 1.21).	Low
**Kent, 2012. Ireland** [49]								
To examine the impact of a SFL on emergency hospital admissions with pulmonary illness among individuals of working age	Comprehensive *Ban*: March 2004	*Pre-ban*: 2002–2003 *Post-ban*: 2005–2006	Pre-posttest non-experimental design	286,000 individuals between the ages of 20 and 69	Emergency medical admissions with acute exacerbations of asthma (ICD-9 and ICD-10)	Hospital In-Patient Enquiry (HIPE) database and census data	Significant reductions were observed in admissions due to asthma (unadjusted RR 0.64; p 0.0001, adjusted by age RR 0.60, in 30–39 years old). These changes remained significant following incorporation of confounding factors into the regression model. The observed changes in admission incidence were influenced by age.	Moderate
**Landers, 2014. US**: Arizona, Colorado Florida,Hawaii, Iowa, Maryland, New Jersey, New York, Rhode Island, Utah,Vermont, Washington, Arkansas, Kentucky, Michigan, South Carolina, Wisconsin [51]								
To examine the relationship between SFL and asthma discharges	Comprehensive	2002–2009 Different locations and different pre and post-ban periods	Pretest-posttest quasi-experimental design	US population (103,000,000 individuals, 35% of the US population). Adults and children admitted for asthma in 17 states (12 state SFL, and 5 states without state SFL as a control group)	Asthma discharges per children or working-age adult. Appendicitis as a control variable.	Healthcare cost and Utilization Project state inpatient data. American Nonsmokers Rights Foundation SFL database	There was a statistically significant relationship (b –2.44) between the implementation of county laws and reductions in workin-age adult asthma discharges. There was no statistically significant effect of state SFL on working-age adult asthma discharges besides the effect of county laws. There was also a statistically significant relationship between the implementation of county some-free laws and reductions in child asthma discharges (b –1.32;) but there was no statistically significant effect of state laws on child asthma discharges besides the effect of county laws.	Low
**Mackay, 2010. Scotland** [35]								
To determine whether the SFL in Scotland, influenced the rate of hospital admissions for childhood asthma and deaths before arrival at the hospital.	Comprehensive *Ban*: 26 March 2006	*Pre-ban*: January 2000 –March 2006 *Post-ban*: March 2006 October 2009	Non-experimental time series design	Children younger than 15 years. Of the 21,415 admissions for asthma, 11,796 (55.1%) occurred among preschool and 9619 (44.9%) among school-age children	Hospital admissions for asthma (ICD-10 J45 or J46)	Scottish Morbidity Record and death-certificate	Before the SFL, admissions for asthma were increasing at a mean rate of 5.2% per year. Post-ban there was a mean reduction in the rate of admissions of 18.2%, per year relative to the ban (p<0.001), net reduction 13% per year(after adjusting for confounders 15.1%) among both preschool and school-age children. There were no significant interactions between admissions for asthma and age group, sex, urban or rural residence, region, or quintile of socioeconomic status. Only 5 deaths occurred over the study period.	Low
**Millet, 2013. England** [59]								
To assess whether the implementation of SFL was associated with a reduction in hospital admissions for childhood asthma and to examine whether changes differed by socioeconomic status (SES).	Comprehensive *Ban*: 1 July 2007	*Pre-ban*: April 2002 *Post-ban*: November 2010	Non-experimental time series design	All children (aged ≤14 years) having an emergency hospital admission for asthma. 217381 admissions, 50.1% preschool (49.9% school). 63.4% in boys. 86.5% in urban locations.	All non- planned admissions with a principal diagnosis of asthma (ICD codes: J45 or J46)	Hospital Episodes Sta- tistics data and census data	Before the implementation of the SFL the admission rate for childhood asthma was increasing by 2.2% per year. After SFL, there was a significant immediate change in the admission rate of -8.9% and change in time trend of 23.4% per year. This change was equivalent to 6802 fewer hospital admissions in the first 3 years after SFL. There were similar reductions in asthma admission rates among children from different age, gender, and socioeconomic status groups and among those residing in urban and rural locations.	Low
**Moraros, 2010. US.** Delaware [60]								
To examine and determine the effects of a comprehensive SFL on the hospitalization rates of patients due to asthma among residents and non-residents	Comprehensive *Ban*: November 2002	*Pre-ban*: 1999–2002 *Post-ban*: 2003–2004	Quasi experimental time series design	Delaware population>18: 783,600 in 2000. 51.4% female. Percentage of smokers: 25.4% in 1999 and 24.4% in 2004. 13.0% ≥65 years.	State and non-state residents discharged with primary discharge diagnosis asthma patients (ICD-9- 410 and 493)	Delaware Department of Health and Social Services. US census data	After adjusting for population growth, the RR for asthma in Delaware residents post-ordinance was 0.95 (0.90 to 0.999), which represented a significant reduction. By comparison, non-Delaware residents had an increased RR for asthma post-ordinance of 1.62 (p< 0.0001).	Low
**Naiman, 2010. Canada**. Toronto [61]								
To study rates of hospital admission attributable to asthma after the implementation of smoking bans.	Partial in three phases: *Ban 1*: Oct 1999 *Ban 2*: June 2001(restaurants) *Ban 3*: June 2004 -March 2006.	*Pre-ban*: 1996 (three years before) *Post-ban*: 1999–2006	Quasi experimental time series design	Toronto: population of about 2.5 million people. Study population = younger than age 65 years.	Admission to hospital diagnostic for asthma (ICD-9 493 and ICD-10 J45 or J46) Compared to control diseases: acute cholecystitis, bowel obstruction and appendicitis	Canadian Census. Database of the Canadian Institute for Health Information	Reduction in rate of admission for asthma only were significant when they compared smoking ban in restaurant vs in public places and workplaces(-0.354, p<0.001)	Moderate
**Rayens, 2008. US.** Kentucky [64]								
To evaluate the effects of a smoke-free law on the rate of ED visits for asthma.	Comprehensive *Ban*: 27 April 2004	*Pre-ban*: 1 January 2001 (40 months before) *Post-ban*: 31 December 2006	Non-experimental time series design	All 5 Lexington-Fayette County hospitals population. The prelaw cohort (262,186 individuals): mean age 29.5, 63% female, Postlaw: mean age 29.7. 63% female	Asthma ED visits, primary and secondary diagnoses (ICD 9 493)	Hospital registers. The 2000 US Census	A total of 14,839 ED visits for asthma events: 7763 prelaw and 7076 postlaw. 36% of prelaw cases were < 20 years, equivalent to the percentage of pediatric cases postlaw. ED visits for asthma increased frequency of over time, also reflected in the age-adjusted rates. Adjusting for confounders, ED visits for asthma declined 22% from prelaw to postlaw (p<0.0001).The rate of decline was 24% in adults≥20 (p 0.0001), whereas the decrease among children 19 years or younger was 18% (p 0.01)	Moderate
**Roberts, 2012. US**. Rhode Island [66]								
To determine whether Rhode Island’s SFL reduced hospital admission rates and associated costs for asthma.	Comprehensive *Ban*: March 2005	*Pre-ban*: 2003–2004 *Post-ban*: Phase I: 2006–2007 Phase II: 2008–2009	Non-experimental time series design	Adults> = 18 years residents in Rhode Island’s. Population size not shown	Admissions to one of Rhode Island’s 11 acute care general hospitals for asthma (ICD-9 493), appendicitis as the control condition	Rhode Island’s Hospital Discharge Data	There was a significant increase in hospitalization rates for asthma between 2003 (11.3) and 2009 (13.5).	Moderate
**Sims, 2013. England** [53]								
To investigate if SFL was associated with an immediate reduction in hospital admissions for asthma in adults and whether any association differs across regions	Comprehensive *Ban*: 1 July 2007	*Pre-ban*: April 1997 *Post-ban*: December 2010	Non-experimental time series design	England residents adults ≥ 16 years (43 million individuals).	Emergency admissions for adult asthma (primary diagnosis, ICD-10 code J45 and J46)	Hospital Episode Statistics data	502 000 emergency admissions had a primary diagnosis of asthma in the period 1997–2010. SFL was associated with an immediate 4.9% (0.6 to 9.0) reduction in emergency admissions for asthma in the adult population. Approximately 1900 emergency admissions for asthma were prevented in each of the first three years after SFL. The reduction in admissions did not vary significantly across regions.	Moderate
**Yildiz, 2015. Turkey.** Kocaeli [75]								
To evaluate admissions to ED for smoking-related diseases prior to and following the introduction of SFL in Kocaeli.	Comprehensive *Ban*: 19 July 2009	*Pre-ban*: Jan 2009 *Post-ban*: June 2010	Non-experimental time series design	Patients visiting ED of 13 hospitals. Kocaeli. Population size not shown.	Emergency visits for asthma (ICD-10 J45), nasopharyngitis, rinitis, allergic rhinitis (ICD J.30),	Directorate of Health in Kocaeli	Total admissions for smoking-related diseases were 83089 in 2009 and 64314 in 2010, a 22.6% decrease. The number of patients admitted with asthma showed a non-significant increase (Increase nº 6805 to 7895)	Moderate
**COPD**								
**Croghan, 2015. US.** Minnesota (Olmsted County) [34]								
To evaluate the impact of the implementation of a SFL on the frequency of ED visits for COPD	Comprehensive Ban: 16 May 2007. Enacted on 1 October 2007	*Pre-ban*: 1 Jan 2005 *Post-ban*: 31 Dec 2009	Non-experimental time series design	2013 population, 147,066 (86.5% white, 51.1% female), median age 75 for COPD; 47 for adults.	ED visits for primary diagnosis of COPD (ICD, 9th codes 491–492 and 494–496)	Mayo Clinic and Olmsted Medical Center	5,293 ED visits occurred with a primary diagnosis of COPD during the 5-year study period. Not significant reduction was detected in COPD-related ED visits following the enactment of the SFL.	Low
**Dusemund, 2014. Switzerland** [38]								
To evaluate the effect of the SFL on the incidence of hospital admissions for acute exacerbation of COPD (AECOPD)	Comprehensive *Ban*:1 March 2008	*Pre-ban*:1 March 2003 2008 *Post-ban*: 28 Feb 2010	Pretest-posttest quasi-experimental design.	Inhabitants of the canton of Graubünden (GR; 191,988) vs the rest of Swittzerland (CH, 7,272,481)	Admissions for AECOPD (ICD-10-codes: J40–44).	Nation-wide database (all hospitalizations in Switzerland)	After the introduction of the SFL and despite clear seasonal variations, the incidence of AECOPD decreased 22.4% in hospitalizations in GR (p <0.001). In the same period, the incidence of AECOPD hospitalizations only slightly decreased by 7.0% in the rest of CH, p<0.001.	Low
**Gaudreau 2013. Canada** Prince Edward Island (PEI) [42]								
To examine changes in hospital admission rates for COPD before and after a SFL.	Comprehensive *Ban*: 1 June 2003	*Pre-ban*: April 1995 -May 2003 *Post-ban*: June 2003—December 2008	Quasi-experimental time series design	PEI population of 143,000. Province of New Brunswick (NB) population 729,995.	Admission for COPD (ICD-9 491, 492, 494, 496 and ICD-10 J41-J44) restricted to ≥35	PEI acute care hospitals registers. Census data from 2001 and 2006	Among all hospital admissions for COPD, males were 57.3%. COPD admissions peaked at 75 to 84 years of age (35.7%). COPD admissions showed a non-significant decrease in mean monthly admission rates immediately after the 2003 SFL. Change in monthly rates means of admission after the SFL by sex, per 100,000 population 1995 to 2008: COPD male -11.79(-32.51, 8.93), female 1.67(-18.84, 22.17). And by age group: COPD 35–64 years:0.64 (-11.12 to 6.79), 65–104 years: 0.94(-56.82 to 52.32)	Low
**Head, 2012. US.** Beaumont (Texas) [45]								
To examine hospital discharge data on 5 tobacco-related diagnoses before and after implementation of a SFL	Comprehensive *Ban*: July 2006	*Pre- Ban*: July 2004-June 2006. *Post Ban*: July 2006-June 2008	Pre-posttest quasi-experimental design	All residents. Intervention city: Beaumont (≈115,000). Control city:Tyler (≈ 87,600). Mean age 33 years, Beaumont non-Hispanic black population 50%	Hospital discharge rates for COPD (ICD-9 491,492, 496)	Texas Department of State Health Services Discharge. Census information	Discharge rates in the intervention city (Beaumont) declined significantly for COPD (RR, 0.64) for whites only. Discharge rates for COPD in the control city (Tyler) did not change.	High
**Humair, 2014. Switzerland.** Canton of Geneva [48]								
To evaluate the effect of the public smoking ban on hospital admissions for acute respiratory diseases	Comprehensive *Ban*: 1 July 2008	*Pre-ban*: 1 July 2006–1 July 2008 *Post-ban*: 1rst ban: 1 July 2008–30 Sept 2008 No ban: 1 Oct 2008–31 Oct 2009 2nd Ban: 30Oct 2009-Dec 2010	Pre-posttest non experimental design	Patients aged ≥ 16 admitted to University Hospitals of Geneva (≈450,000 inhabitants). 5345 total admissions: 60% males, mean age 67. 436 first AECOPD	First hospitalization for COPD entities among chronic lower respiratory diseases (ICD-10 codes: J40–44)	Hospital database of the University Hospitals of Geneva. Census data of Geneva	For AECOPD, the weekly number of hospitalizations dropped over the 4 periods from 2.45 to 1.54 (p 0.0001).The adjusted IRR decreased significantly in all periods after the initial smoking ban; it reached 0.54 for all patients and 0.53 for Geneva residents during the 2nd ban. There was a change in the slope of the trend line across the study periods. The reduction started before the legislative smoking ban and became stable in the second part of the period with no ban but partially maintained in practice. The smoking ban could prevent yearly 47 new hospitalizations for AECOPD.	Moderate
**Kent, 2012. Ireland** [49]								
To examine the impact of a SFL on emergency hospital admissions with COPD	Comprehensive *Ban*: March 2004	*Pre-ban*: 2002–2003 *Post-ban*: 2005–2006	Pre-posttest non experimental design	286,00 individuals of working age: 20–69 years	Emergency medical admissions with exacerbations of COPD (ICD-9 and ICD-10)	Hospital In-Patient Enquiry database. Census data	COPD admissions increased in unadjusted analysis (unadjusted RR, 1.21; p 0.04), but significance was lost in adjusted analysis (adjusted RR, 1.18, p.30). The observed changes in admission incidence were markedly influenced by age.	Moderate
**Naiman, 2010. Canada**. Toronto [61]								
To study rates of hospital admission attributable to COPD after the implementation of smoking bans.	Partial (3 phases) *Ban 1*: October 1999 *Ban 2*: June 2001(restaurants) *Ban 3*: June 2004-March 2006	*Pre-ban*: 1996 (three years before) *Post-ban*: 1999–2006	Quasi experimental time series design	Toronto: population of about 2.5 million people. Study population = younger than age 65 years.	Admission to hospital diagnostic for COPD (ICD-9 433–436 and ICD-10 I63-66 or G45-46). Compared to control disease: acute cholecystitis, bowel obstruction and appendicitis	Canadian Census. Database of the Canadian Institute for Health Information	Reduction in rate of admission for COPD only were significant when they compared smoking ban in restaurant vs in public places and workplaces(-1.040, p<0.008)	Moderate
**Vander, 2012. US** [54]								
To examine effects of 3 types of smoking bans -in restaurants, bars, and workplaces-to determine whether their impact differs according to location and whether there is a relationship between the comprehensiveness and COPD admissions (vs non SHS related diseases)	Different types of laws in different places. 938 laws passed by municipalities, counties, and states to ban smoking in workplaces, restaurants, and bars	1991–2008. *Pre-ban*: no specified *Post-ban*: 1–3 months after 4–12 months after 13–36 months after >36months after	Pretest-posttest quasi-experimental design	Medicare beneficiaries age ≥ 65 for counties that ever or never had a smoking ban as of 2008 (≈ 36.5 millions individuals)	Hospital admissions for COPD (ICD-10)	US Tobacco Control Laws Database. 2000 census. Centers for Medicare and Medicaid Services Denominator	Unadjusted admission rates for COPD during 18-year study period increased. By 2008, mean unadjusted admission rates had increased by 80%.There were no significant differences during 1991 in admission rates between counties that ever or never had a ban. Admission rates fell 11% where workplace smoking bans were in place and 15% where bar smoking bans were present. The increase for counties with a new ban was 5% lower than expected within the first 3 months after the first ban, and 10% and 17% lower within 12 months and after 36 months, respectively. Counties with bans in one, two, or three settings experienced increases in COPD admission rates that were 9%, 14%, and 7% lower than those experienced by counties without bans, respectively (p< 0.001)	High
**Yildiz, 2015. Turkey.** Kocaeli [75]								
To evaluate admissions to ED for smoking-related diseases prior to and following the introduction of SFL	Comprehensive *Ban*: 19 July 2009	*Pre-ban*: Jan 2009 *Post-ban*: June 2010	Non-experimental time series design	Patients visiting ED of 13 hospitals. Kocaeli. Population size not shown.	Emergency visits for COPD (ICD-10 J.44)	Directorate of Health in Kocaeli	There was a large decrease in the numbers of patients admitted to emergency departments with COPD after the smoking legislation was introduced than before (8342 versus 6571, p>0.05)	Moderate
**Pneumoniae/Bronchitis**								
**Humair, 2014. Switzerland.** Canton of Geneva [48]								
To evaluate the effect of the public smoking ban on hospital admissions for acute respiratory diseases	Comprehensive *Ban*: 1 July 2008	*Pre-ban*: 1 July 2006–1 July 2008 *Post-ban*: 1rst ban: 1 July 2008- Sept 2008 No ban: Oct 2008–31 Oct 2009 2nd Ban: 30 Oct 2009-Dec 2010	Pre-posttest non-experimental design	Pacients aged ≥ 16 admitted to University Hospitals of Geneva, (≈450,000 inhabitants).5345 admissions: 60% males, mean age 67. 239 firsts pneumonia	First hospitalization for pneumonia or influenza (ICD-10 codes: J 10–16)	Hospital database of the University Hospitals of Geneva. Census data of Geneva	Despite variations in the number of admissions, adjusted IRR of hospitalizations for pneumonia did not significantly change throughout the 4 periods. IRR all patients for the final period 1(0.75–1.35) and for Geneva residents only 0.97(0.70–1.35)	Moderate
**Kent, 2012. Ireland** [49]								
To examine the potential impact of SFL on emergency hospital admissions with pulmonary illness	Comprehensive *Ban*: March 2004	*Pre-ban*: 2002–2003 *Post-ban*: 2005–2006	Pre-posttest non-experimental design	286,000 individuals of working age:20–69 years.	Emergency medical admissions with acute pneumonia, lower respiratory tract infection-LRTIs (ICD-9 and ICD-10)	Hospital In-Patient Enquiry database. Census data	Significant reductions were observed in admissions due to pneumonia (unadjusted RR, 0.77; 0.64 to 0.93; adjusted 0.71, 0.52–0.98). These changes remained significant following incorporation of confounding factors into the regression model. The observed changes in admission incidence were markedly influenced by age.	Moderate
**Naiman, 2010. Canada**. Toronto [61]								
To study rates of hospital admission attributable to pneumonia or bronchitis after the implementation of smoking bans.	Partial (3 phases) *Ban 1*: October 1999 *Ban 2*: June 2001(restaurants) *Ban 3*: June 2004 to March 2006).	*Pre-ban*: 1996 (three years before) *Post-ban*: 1999–2006	Quasi experimental time series design	Toronto: population of about 2.5 million people. Study population = younger than age 65 years.	Admission to hospital diagnostic for pneumonia or bronchitis (ICD-9 266, 480–486 and ICD-10 J12-18, J20). Compared to control disease: acute cholecystitis, bowel obstruction and appendicitis	Canadian Census. Database of the Canadian Institute for Health Information	Crude rates of admission to hospital because of conditions decreased by 33% (32% to 34%) during the ban period affecting restaurant settings (no when affecting other settings). There was a 13.5% overall reduction in admissions for respiratory conditions (p 0.239). Reduction in rate of admission for pneumonia or bronquitis only were significant when they compared smoking ban in restaurant vs in public places and workplaces(-0.598)	Moderate
**Yildiz, 2015. Turkey.** Kocaeli [75]								
To evaluate admissions to ED for smoking-related diseases prior to and following the introduction of SFL	Comprehensive *Ban*: 19 July 2009	*Pre-ban*: January 2009 *Post-ban*: June 2010	Non-experimental time series design	Patients visiting emergency department of 13 hospitals. Kocaeli. Population size not shown.	Emergency visits for bronchitis (ICD-10 J.20)	Directorate of Health in Kocaeli	Total admissions for smoking-related diseases were 83089 in 2009 and 64314 in 2010, a 22.6% decrease. The number of patients who were admitted to the emergency department with chronic bronchitis was 44141 for the 6-month period in 2009 and 26558 over the same 6-month period in 2010, a reduction of 39.8%(p<0.01).	Moderate
**Pneumothorax**								
**Kent, 2012. Ireland** [49]								
To examine the impact of SFL on emergency hospital admissions with pulmonary illness	Comprehensive *Ban*: March 2004	*Pre-ban*: 2002–2003 *Post-ban*: 2005–2006	Pre-posttest non-experimental design	286,000 individuals of working age: 20–69 years	Admissions for spontaneous pneumothorax (ICD-9 and ICD-10)	Hospital In-Patient Enquiry database. Census data	No significant change was observed in unadjusted or adjusted analysis with regard to emergency admissions due to spontaneous pneumothorax (RR 0.62). The observed changes in admission incidence were markedly influenced by age.	Moderate
**Lower respiratory infection**								
**Kent, 2012. Ireland** [49]								
To examine the potential impact of the Irish smoking ban on emergency hospital admissions with pulmonary illness	Comprehensive *Ban*: March 2004	*Pre-ban*: 2002–2003 *Post-ban*: 2005–2006	Pre-posttest non-experimental design	286,000 individuals of working age: 20–69 years.	Emergency admissions for exacerbations LRTIs (ICD-9 and ICD-10)	Hospital In-Patient Enquiry database. Census data	Admissions due to acute pulmonary illness declined significantly from 439 admissions per 100,000 population per annum in pre-ban period to 396 in post-ban period (unadjusted RR 0.91, adjusted 0.83). This decrease persisted following adjustment for confounding variables (RR, 0.85). No significant change was observed in emergency admissions due to lower respiratory tract infections/acute bronchitis.	Moderate
**Yildiz, 2015. Turkey.** Kocaeli [75]								
To evaluate admissions to ED for smoking-related diseases prior to and following a SFL	Comprehensive *Ban*: 19 July 2009	*Pre-ban*: Jan 2009 *Post-ban*: June 2010	Non-experimental time series design	Patients visiting ED of 13 hospitals in Kocaeli. Population size not shown.	Emergency visits for bronchitis (J.20), LRTI/pneumonia (J.22/J.18)	Directorate of Health in Kocaeli	Total admissions for smoking-related diseases were 83089 in 2009 and 64314 in 2010, a 22.6% decrease. Time-series analysis showed that the decreases were significant for bronchitis and lower respiratory tract infections.	Moderate
**Respiratory diseases**								
**Dilley, 2012. Washington State** [36]								
To examine health effects associated with 3 tobacco control interventions: a comprehensive state program, a state policy banning smoking in public places, and price increases	Comprehensive *Ban*: December 2005	*Pre-ban*: 1990 *Post-ban*: December 2008	Non-experimental time series design	Washington State adults’. Population size not shown.	Hospitalization diagnosis for COPD, emphysema, asthma, chronic bronchitis (ICD-9 490–496), lung, bronchus and trachea cancer(ICD-9 162)	State’s Surveillance and Survey. Hospital Reporting System. State’s cancer registry	Smoking declines in the state exceeded declines in the nation. The state program had the most consistent and largest effect on trends for respiratory disease. Policy effect was less often negative (4 of 7 hospitalization-incidence models) but infrequently significant (1 of 7 hospitalization-incidence models). Chronic respiratory disease hospitalizations: Policy effect R2 with national adjustment = 2.13, p 0.79, R2 without national adjustment = 1.16, p 0.89	Low
**Kent, 2012. Ireland** [49]								
To examine the impact of SFL on emergency hospital admissions with pulmonary illness	Comprehensive *Ban*: March 2004	*Pre-ban*: 2002–2003 *Post-ban*: 2005–2006	Pre-posttest non-experimental design	286,000 individuals of working age: 20–69 ys.	Emergency admissions for exacerbations LRTIs (ICD-9 and ICD-10)	Hospital In-Patient Enquiry database. Census data	A significant reduction in overall pulmonary admissions was observed in the 20- to 29-year-old age group (adjusted RR, 0.62), with a similar trend in 30- to 39-year-olds (adjusted RR, 0.74). No significant reduction was seen in older age groups.	Moderate
**McGhee, 2014. China.** Hong Kong [80]								
To examine effects on hospital admissions for conditions associated with SHS following a smoke-free workplace legislation	Partial *Ban*: 27 October 2006. Law extended on 1 January 2007	*Pre-ban*: 1997–2006 *Post-ban*: January 2007 to 2008	Pre-posttest non-experimental design (with control diseases)	2013 general population. Population size not shown.	Hospital admissions for respiratory condition (ICD-9-CM 460–519), lung cancer (ICD-9-CM 162)	Hospital Authority Clinical Management System	A seasonal peak in hospital admissions for respiratory disease in all ages was reduced from 12.6% to 10.1% in the first year after intervention; however, there was a rebound to 12.9% in the second year.	Moderate

AECOPD: acute exacerbated chronic obstructive pulmonary disease. AMI: acute myocardial infarct. ARIMA: Autoregressive Integrated Moving Average. CH: Switzerland. COPD: chronic obstructive pulmonary disease. ED: Emergency department. GR: Graubünden. IRR: incidence rate ratio. LRTIs: lower respiratory tract infections. NHANES: National Health and Nutrition Examination Survey. NB: New Brunswick. PEI: Prince Edward Island. R2: Coefficient. RR: rate ratio. SE: standard error. SES: socioeconomic status. SFL: Smoke free laws/legislation. SUAT: Servicio de Urgencia, Asistencia y Traslado. US: United States

**Table 3:** Summary of studies on the impact of smoke-free legislation on mortality.

Aims	Legislation	Study period	Study design	Study participants and size	Variables	Source of information	Summary of findings	Risk of bias
**Binswanger, 2014. United States (US)** [33]								
To determine whether bans on smoking in prison are associated with reductions in smoking related deaths.	Different types of laws. In 2001, half of states had any smoking ban (n = 25). By 2011, 48 states had a ban on smoking in prison	From 2001 to 2011	Quasi- experimental time series design	All state prisons in the US. 14,449 individuals. 49.6% of people in 2004 were aged ≥35, and 6.8% were women. Among people of all ages, 75.8% had ever smoked.	Rates of smoking related deaths, including from cancer, cardiovascular disease, and pulmonary disease	Web based searches of state policies and legislation. Bureau of Justice Statistics	Any ban was associated with a reduced incidence of any smoking related death (adjuste IRR 0.91), including significant reductions in pulmonary deaths (0.71). Men had significantly higher rates of death than women for all smoking related causes and cancer. Bans in place for more than nine years were associated with significant reductions in all smoking related deaths (IRR 0.89), cancer deaths (IRR 0.81), and pulmonary deaths (IRR 0.66) compared with places with no ban	Moderate
**McGhee, 2014. China.** Hong Kong [80]								
To examine effects on trends in deaths for conditions associated with SHS following a smoke-free workplace legislation	Partial *Ban*: 27 Oct 2006. Law extended on 1 January 2007	*Pre-ban*: 1997–2006 *Post-ban*: Jan 2007 to 2008	Pre-posttest non-experimental design (with control diseases)	2013 general population. Population size not shown.	Deaths for respiratory condition (ICD-9-CM 460–519), lung cancer (ICD-9-CM 162)	Hospital Authority Clinical Management System. Census data	The annual proportional changes in mortality were significant in lung cancer (which decreased among all ages): relative changes -5.65, authors suggested that this is not attributable to the SFL, but to improved treatment and other factors as follow-up	Moderate
**Stallings-Smith, 2013. Republic of Ireland** [70]								
To assess the effect of a SFL on all-cause and cause-specific, non-trauma mortality	Comprehensive *Ban*: 29 March 2004	*Pre-ban*: 1 January 2000 *Post-ban*: 31December 2007	Non-experimental time series design	Irish population, ages ≥35 years. 1.9 million individuals.	Death for all respiratory diseases (460-519/J0-J99) and COPD (490–492, 494–496/J40–J44, J47).	Mortality data. Irish Health Protection Surveillance Centre for influenza data	Mortality decreases were primarily due to reductions in passive smoking. All respiratory female immediate effects 0.64 (0.42–0.98). Post-ban, a 38% reduction in COPD mortality was observed. Post-ban reductions in COPD mortalities were seen in ages ≥65 years, but not in ages 35–64 years. COPD mortality reductions were found only in females (RR: 0.47), COPD overall 0.62, females immediate effects 0.47, COPD ages 65–84 immediate effects 0.68, ages≥85 years immediate effects 0.49	Low
**Stallings-Smith, 2014. Republic of Ireland** [69]								
To assess the effects of a SFL on COPD mortality by discrete and composite socioeconomic status (SES) indicators to determine impacts on inequalities.	Comprehensive *Ban*: 29 March 2004	*Pre-ban*: 2000 *Post-ban*: 2010	Non-experimental time series design	Irish population, ages≥35 years. Population size not shown.	Deaths for COPD (490–492, 494–496/J40–J44, J47).	Census data. Mortality data. Irish Health Protection Surveillance Centre for the influenza data	From 2000–2010, total deaths due to COPD (n 15192). Post-ban mortality reductions by structural SES indicators were concentrated in the most deprived tertile: RR≈ 0.66 in low education, RR≈0.62 for non-Irish Nationality. RR≈0.75 for population unemployement. RR≈0.72 for rented/free housing tenure. RR≈0.69 for no car access	Low

COPD: Chronic obstructive pulmonary disease. IHD: Ischemic heart disease. IRR: incidence rate ratio. RR: rate ratio. SES: Socioeconomic status.US: United States

### SFL effect on respiratory symptoms

Of the 50 papers, 26 (52%) evaluated respiratory symptoms. Of these 26, 23 (88.5%) concerned workers, 22 (84.6%) were non-experimental, and 14 (53.8%) assessed comprehensive SFL. Evaluation periods were from one month to six years. The outcomes included: any respiratory symptom (17 studies, 65.4%), wheezing (16 studies, 61.5%), phlegm (14 studies, 53.8%), morning cough (ten, 38.5%), cough during the rest of the day (17 studies, 65.4%), breathlessness (seven, 26.9%), tightness in chest (four, 15.4%) and asthma symptoms (two, 7.7%)[37,58].

The majority of the outcomes presented a post-ban decrease period in the percentage of adults suffering from them. Those that decreased significantly in most of the studies were “any respiratory symptom” (range 7.7 to 42.0%), “morning cough” (4.4 to 30.0%), “coughing the rest of the day” (2.3 to 41.2%), and phlegm (3.5 to 42.0%). In comparison with partial SFL comprehensive SFL appears to produce the greater decline. Six studies reported post-ban decreases in comprehensive SFL for the outcomes breathlessness (range 6.2 to 25%) [32,40,67,76] and tightness in chest (10% of decrease)[67], however, along with wheezing (five papers with significant decreases[32,39,40,55,76] and five with non significant decreases[41,43,44,67,73]) and asthma[58], findings did not indicate a clear decline in these symptoms.

In addition, two studies focused on children in environments with non strictly comprehensive legislations (one with mixed types of SFL in the same paper[37] and one with partial SFL[47]). They showed discrepant results: an increase of 1.9%[47] in any respiratory symptom in the post-ban period and a decrease in asthmatic symptoms, persistent wheezing, and chronic night cough[37].

Effects regarding “any respiratory symptom” appeared to be more intense in the period immediately following implementation of the smoking law (maximum decrease of 42.0%[39]) than six months later (maximum 25.0%[43]), particularly in the studies that evaluated comprehensive SFL. In contrast, in partial SFL the declines in percentages of individuals with “any respiratory symptom” were similiar (around 15.0%[65,74]) irrespective of the moment of evaluation.

**Fig 2:**
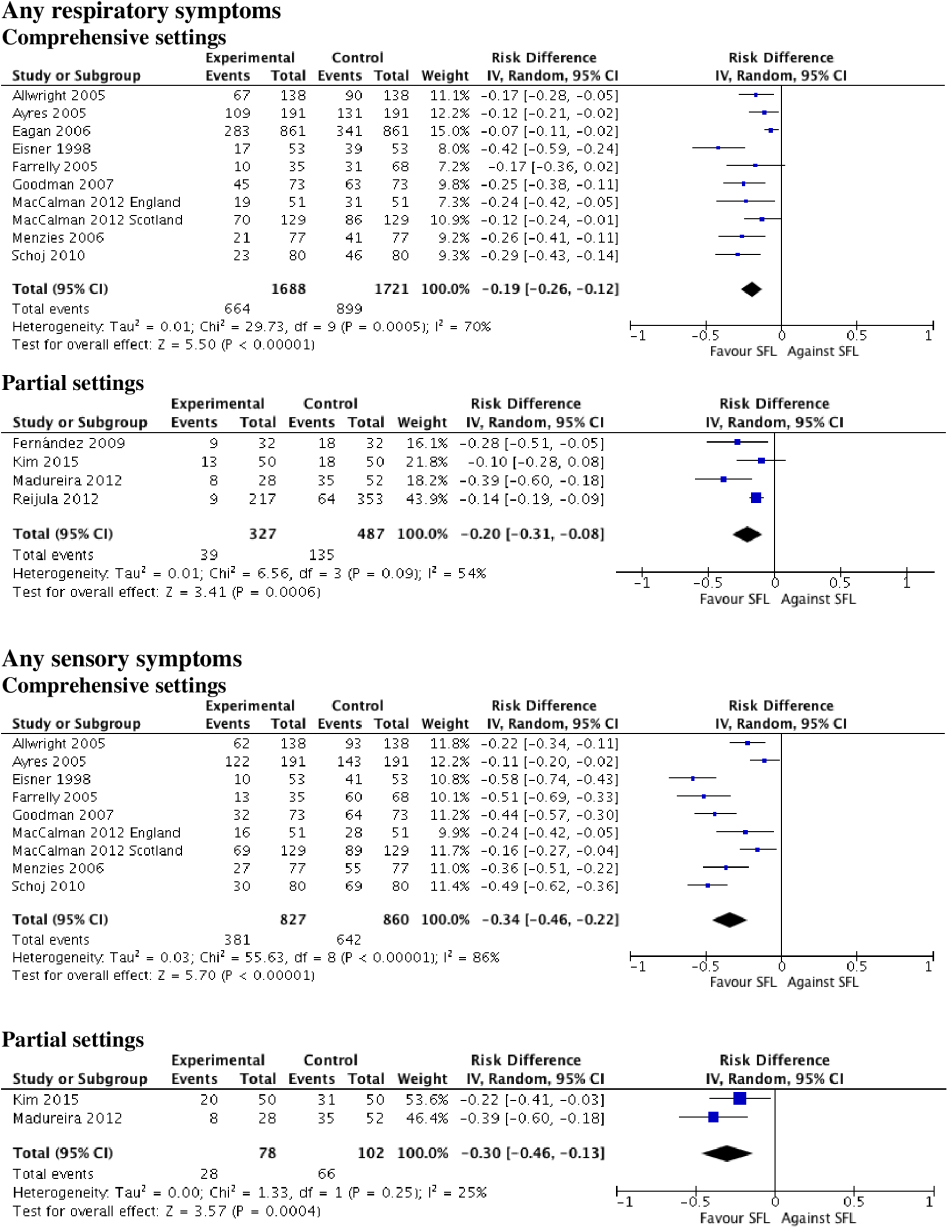
Risk difference between before and after the smokefree legislation (SFL) in any respiratory/sensory symptom. Abbreviations: CI, confidence interval; df, degrees of freedom; IV, Inverse Variance method.

Ten studies were included in the meta-analysis for the outcome “any respiratory symptom” in comprehensive SFL setting (one study was stratified in two due to two regions being analyzed[55][56]). The pooled data (Fig 2) showed a decline of 19% in any respiratory symptom after comprehensive SFL (overall RD = -0.19, 95% confidence interval [95%CI]) = -0.26; -0.12) with substantial heterogeneity between studies (I2 = 70%, p = 0.0005). In the sensitivity analysis, the exclusion of individual studies did not substantially modify the estimates, with the pooled RDs of any respiratory symptom ranging from -0.18 to -0.21. One study[76] was the principal origin of heterogeneity and showed a minor magnitude of the effect. After excluding it from the analysis, heterogeneity decreased (I2 = 41%, p = 0.10) and the pooled data remained similar (overall RD = -0.21, 95%CI = -0.27; -0.15) (S4 Table). Subgroup analysis by study design did not significantly reduce heterogeneity. Minor heterogeneity was found in studies with a moderate or high RoB (although there were only two in each case) in contrast with those of low risk. With respect to subgroup analysis by follow-up time, studies with 12 or more months were less heterogeneous and the pool effect was smaller than those with a with shorter follow-up (S5 Table). Regarding to any respiratory symptom in a comprehensive SFL setting, the asymmetric funnel plot suggested publication bias (S1 Fig).

Four studies were included in the meta-analysis for the outcome “any respiratory symptom” in a partial SFL setting. The pooled data (Fig 2) showed a decline of 20% in any respiratory symptom after the SFL (overall RD = -0.20, 95%CI = -0.31; -0.08) with wide CI and considerable heterogeneity between studies (I2 = 54%, p = 0.09). In the sensitivity analysis, the exclusion of individual studies substantially modified the estimates, with the pooled RDs of any respiratory symptom ranging from -0.14 to -0.25. One study[56] was the principal origin of heterogeneity and had a greater magnitude of the effect in comparison with the rest of the studies. After excluding it from the analysis heterogeneity decreased (I2 = 0%, p = 0.45) and the pooled data was lower than in the previous analysis (overall RD = -0.14, 95%CI = -0.19; -0.10) (S4 Table). Subgroup analysis by study design did not significantly reduce heterogeneity. Minor heterogeneity was found in studies with moderate RoB, (a small number of studies involved only two) than those with low risk. With respect to subgroup analysis by follow-up time, there was only one study with a short follow-up. Studies with 12 or more months were heterogeneous although the pool effect was greater than the initial analysis (S5 Table).

### SFL effect on sensory symptoms

Of the 50 papers, 19 (38.0%) evaluated the presence of sensory symptoms. Of these 19, all solely focused on workers, 17 (89.5%) were non-experimental, and 11 (57.9%) evaluated a comprehensive SFL effect. Periods of evaluation ranged from one month to two years. Outcomes were: any sensory symptom (15 studies, 78.9%), red/irritated eyes (15 studies, 78.9%), runny nose/sneezing (14 studies, 73.7%), and sore/scratchy throat (13 studies, 68.4%).

All the 15 studies found significant decreases in the number of individuals who had “any sensory symptom” in the before-after comparision (range of decrease 11.0–100.0%) irrespective of the kind of legislation. Twelve studies showed statistically significant decreases for the outcomes “red/irritated eyes” (range 7.5 to 44.0%) and “runny nose /sneezing” (6.3 to 45.0%). The outcome “sore/scratchy throat” decreased non- significantly in four studies[55–57,72], and in the rest of the nine studies it declined in the post-ban period (range 12.0[32] to 48.7%[40]), particularly in those that evaluated comprehensive SFL.

The “any sensory symptom” outcome showed a greater decrease immediately following implementation of legislation (up to a maximum of 70.0–100.0%[62] at six months versus 50% at longer periods[41]), especially in the case of comprehensive SFL, with even higher percentages than respiratory symptoms in studies assessing both respiratory and sensory symptoms.

Nine studies were included in the meta-analysis for the outcome “any sensory symptom” in a comprehensive SFL setting (one study was stratified due to having two regions analyzed[55] [56]). The pooled data (Fig 2) showed a decline of 34% in any sensory symptom after the comprehensive SFL (overall RD = -0.34, 95%CI = -0.26; -0.12) with substanial heterogeneity between studies (I2 = 86%, p < 0.001). In the sensitivity analysis, the exclusion of individual studies did not substantially modify the estimates, with the pooled RDs of any sensory symptom ranging from -0.31 to -0.37. No study was observed to be the main origin of heterogeneity between studies, I2 remained above 80% in the sensitivity analysis (S4 Table). Subgroup analysis by study design did not significantly reduce heterogeneity, although minor heterogeneity was found in studies with a moderate or high RoB (a small number of studies involved only two) than those with low. Regarding subgroup analysis by follow-up time, the studies with a short follow-up had a greater pool effect than those with 12 or more months, marked heterogeneity being maintained in both cases (S5 Table).

Two studies were included in the meta-analysis for the outcome “any sensory symptom” in a partial SFL setting. The pooled data (Fig 2) showed a decline of 30% in any sensory symptom after the SFL (overall RD = -0.30, 95%CI = -0.46; -0.13) with wide CI and low heterogeneity between the studies (I2 = 25%, p = 0.25).

### SFL effect on spirometry parameters

Out of the 50 papers, eight studies (16.0%)[39,43,52,58,67,68,71,78] assessed spirometric parameters in workers. Of these eight, all were non-experimental and five (62.5%) were performed in comprehensive SFL settings[39,43,58,67,68]. Evaluation periods ranged from one month to two years. Of the eight studies, four (50.0%) had an increase in forced expired volume in one second (FEV1)[39,58,68,71], although two of these were carried out with non-smokers[39,68] and asthmatic cohorts[68]. Six studies (75.0%) evaluated forced vital capacity (FVC) which increased significantly (range 3–4.2%) in five[39,43,68,71,78] although in one it was only augmented in an asthmatic cohort [68]. Discrepant results were obtained when the effect of comprehensive SFL on forced mid-expiratory flow rate (FEF_25-75%_)[39,43,68,71] and peak expiratory flow rate (PEF)[43,68] was assessed.

**Fig 3:**
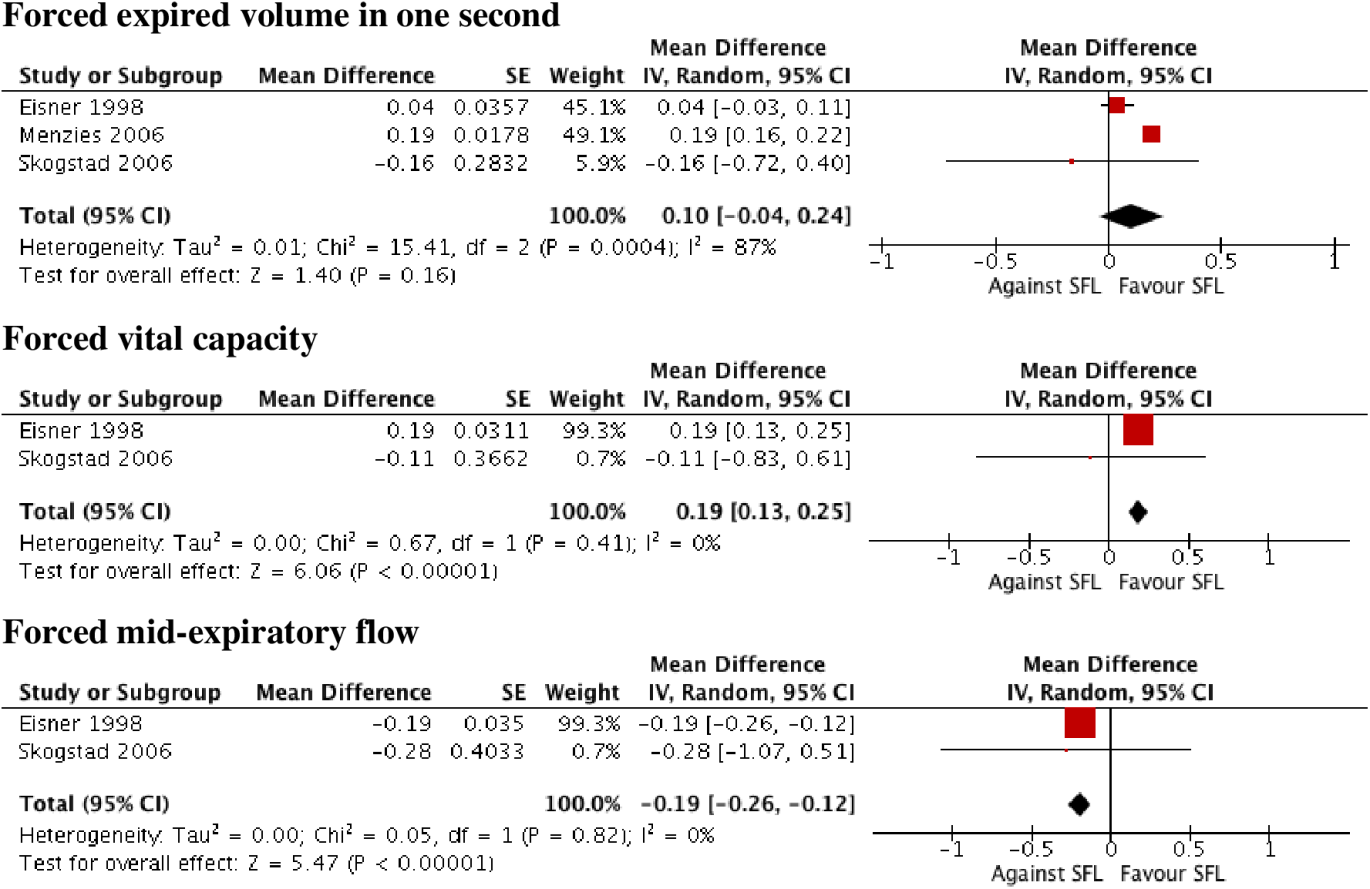
Mean difference between before and after comprehensive smokefree legislation (SFL) in spirometry parameters. Abbreviations: CI, confidence interval; df, degrees of freedom; IV, Inverse Variance method.

Three studies were included in the meta-analysis for the outcome “FEV1” in a comprehensive SFL setting. Pooled results indicated a non significant net difference in FEV1 between before and after comprehensive SFL (overall MD = 0.10, 95%CI = -0.04; 0.24; I2 = 87%) (Fig 3). In the sensitivity analysis, the exclusion of individual studies did not substantially modify the estimates, with pooled MDs of FEV1 ranging from non significant values of -0.04 to 0.13.

Only two studies could be included in the meta-analysis for the outcome “FVC” in a comprehensive SFL setting. Pooled results indicated a significant net difference in FVC between before and after comprehensive SFL (overall MD = 0.19, 95%CI = 0.13; 0.25) with homogeneity (I2 = 0%, p = 0.41) (Fig 3).

Only two studies could be included in the meta-analysis for the outcome “FEF_25-75%_” in a comprehensive SFL setting. Pooled results indicated a significant net difference in FEF_25-75%_ between before and after comprehensive SFL (overall MD = -0.19, 95%CI = -0.26; -0.12) with homogeneity (I2 = 0%, p = 0.82) (Fig 3).

### SFL effect on asthma admissions

Out of the 50 papers, 17 (34%) concerned asthma admissions (all but two in hospital settings). Of these 17, nine (52.9%) were in the general population, five (29.4%) in adults, and three (17.6%) in children. Ten (58.8%) were non-experimental and 15 (88.2%) evaluated comprehensive SFL. One was conerned asthma treatment use (5.9%)[79]. Evaluation periods ranged from 11 months to seven years. Significant decreases were described in 13 of the 17 papers evaluating asthma hospital admissions.

With respect to the nine studies carried out in the general population[34,42,45,46,51,60,61,64,75], six of them (66.7%) were quasi-experimental[42,45,46,51,60,61], and, with the exception of one[61], all were performed in comprehensive SFL locations. In eight studies (88.9%), admission rates for asthma (both hospital and non-hospital admissions) significantly declined with a range of 5.0%[60] to 31.0% (the latter figure was for Caucasians in Texas[45]). In addition, a significant annual rate of reduction of -0.35 [95% confidence interval (CI) -0.53 to -0.018] in hospital asthma admissions was obtained in the sole study on partial SFL[61] when it compared SFL in restaurants versus public areas and workplaces over the ten-year study period. Four stratified studies (44.4%)[34,42,51,64] all reported significant reductions in asthma hospital admissions both in children (range 18.0[64]- 25.0%[34]) and adults (range 16.0[34]-24.0%[64]).

**Fig 4:**
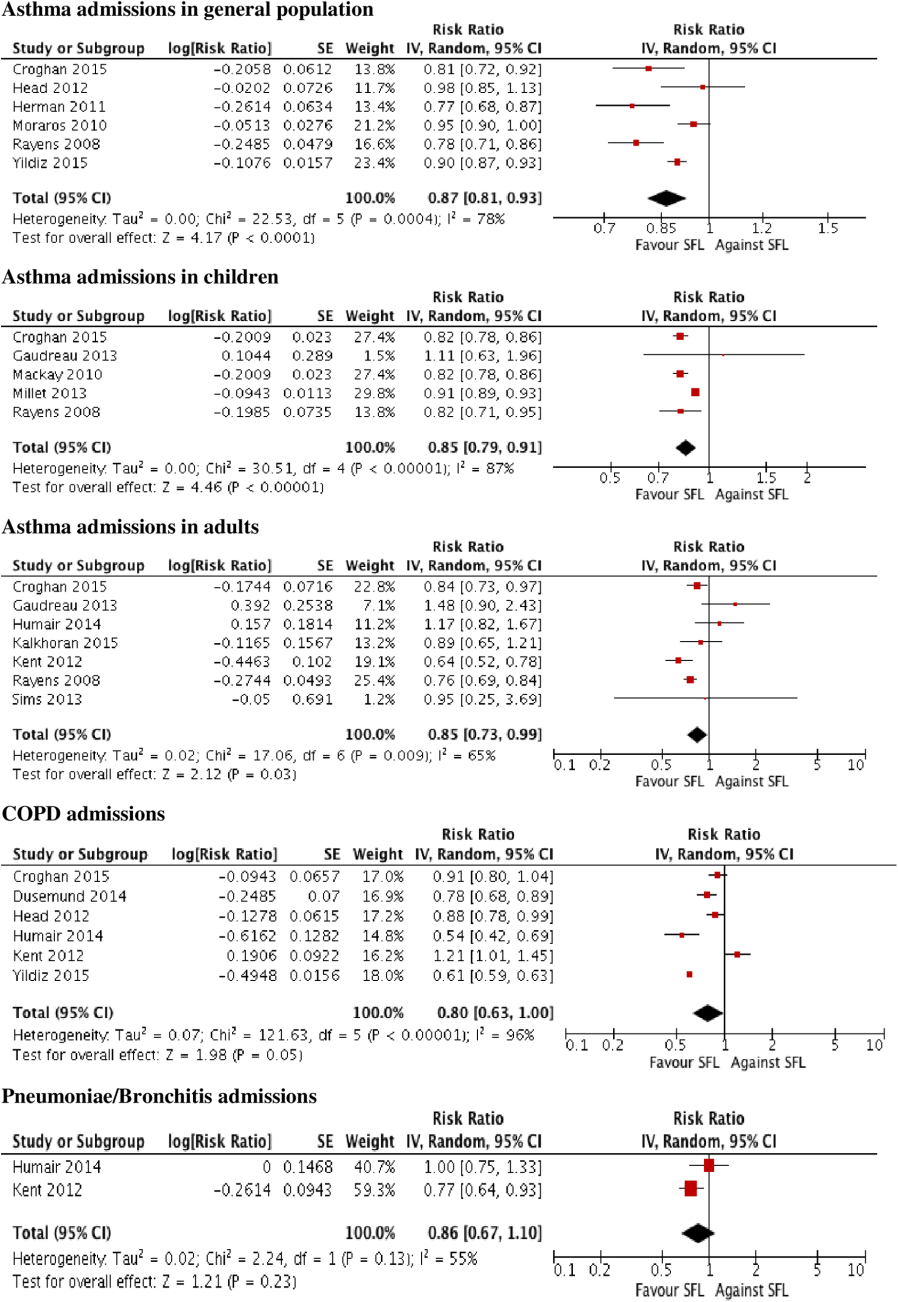
Risk ratio between before and after comprehensive smokefree legislation (SFL) in asthma, COPD and lung infection admissions. Abbreviations: CI, confidence interval; df, degrees of freedom; IV, Inverse Variance method.

Six studies were included in a meta-analysis for the outcome “asthma admission” in a general population in a comprehensive SFL setting (Fig 4). According to the forest plot, there was a significant decrease of 13% after SFL (overall RR = 0.87; 95%CI = 0.81; 0.93). Heterogeneity was high (I2 = 78%, p <0.001). In the sensitivity analysis, the exclusion of individual studies did not substantially modify the estimates, with pooled RRs of asthma admissions ranging from 0.85 to 0.89. No study was found to be the main origin of heterogeneity between studies, I2 remained above 70% in the sensitivity analysis by omitting one at a time (S4 Table). Subgroup analysis by study design, risk of bias and follow-up time (less than 24 months vs 24 months or more) did not significantly reduce heterogeneity (S6 Table).

Three studies were focused on children[35,37,59], in two (66.7%) there were significant reductions of hospital asthma admissions in comprehensive SFL locations (declines of 9.0%[59] and 18.2%[35]) whilst in the remaining paper on regions with different types of SFL there was no significant decrease[37].

Five studies were included in the meta-analysis for the outcome “asthma admission” in children in comprehensive SFL setting (three focused on children and two were stratified by age) (Fig 4). There was a significant decrease of 15% after SFL (overall RR = 0.85, 95%CI = 0.79; 0.91). Heterogeneity was high (I2 = 87%, p <0.001). In the sensitivity analysis, exclusion of individual studies did not substantially modify the estimates, with pooled RRs of asthma admissions ranging from 0.82 to 0.86. One study, which had the lowest RR, was the main origin of heterogeneity between studies[59]. After excluding it from the analysis, the heterogeneity decreased (I2 = 0%, p = 0.77) and the pooled effect was higher than in the previous analysis (overall RR = 0.82, 95%CI = 0.79; 0.84) (S4 Table). Subgroup analysis by study design and RoB did not significantly reduce heterogeneity. All the studies had more than 24 months of follow-up (S6 Table).

Five studies focused on adult populations[48,49,53,66,79], they all evaluated comprehensive SFL and had a non-experimental design. Three of them (60.0%) found significant reduction rates from 4.9%[53] to 36.0%[49] (all except one in hospital admissions[79]). One study reviewed the incidence rate ratio in non-hospital admissions [79] and reported a decrease of 15.0% in admissions after SFL. In the two remaining studies[48,66], results were discordant with no significant differences. With respect to asthma treatment in adults, the use of salbutamol and ipratropium descended in non-hospital emergency settings[79].

Seven studies were included in the meta-analysis for the outcome “asthma admission” in adults in comprehensive SFL setting (five focused on adults and two were stratified by age) (Fig 4). There was a significant decrease of 15% after SFL (overall RR = 0.85, 95%CI = 0.73; 0.99). Heterogeneity was considerable (I2 = 65%, p = 0.009). In sensitivity analysis, exclusion of individual studies modified the estimates substantially, with pooled RRs of asthma admissions ranging from 0.80 to 0.90. Two studies were the main origin of heterogeneity between studies[42,48], they had no significant increases of asthma admissions. After excluding them from the analysis, the heterogeneity decreased (I2 = 31%, p = 0.22) and the pooled data was more robust (overall RR = 0.77, 95%CI = 0.70; 0.85) (S4 Table). Subgroup analysis by study design and RoB did not significantly reduce heterogeneity. With respect to subgroup analysis by follow-up time, only one study had less than 24 months of follow-up, and those with longer periods had a greater pool effect than the initial analysis, maintaining high heterogeneity (S6 Table).

### SFL effect on COPD admissions

Nine (18.0%) of the 50 papers reported effects on COPD admissions[34,38,42,45,48,49,54,61,75](all except one in hospital settings): six (66.7%) in a general population and three (33.3%) in adults exclusively. Five (55.5%) were quasi-experimental and eight (88.9%) evaluated comprehensive SFL. Evaluation periods ranged from 11 months to 5.5 years. Significant decreases were described in 6 of the 9 papers evaluating asthma hospital admissions.

In four (66.7%)[38,42,45,61] of the six articles which focused on the general population and had a quasi-experimental design, significant decreases in COPD admissions, ranging from 1.0%[61] (a study that compared the different phases of SFL before becoming comprehensive) to 36.0%[45], were found. The other two studies (33.3%), with non-experimental design[34,75], presented non-signficant declines.

There were three studies that evaluated comprehensive SFL in adults[48,49,54]. In two of them (66.7%), hospital admissions for COPD decreased significantly (from 15.0%[54] to 46.0%[48]) whilst in the other study (33.3%) they increased non significantly (adjusted relative risk 1.18; 95% CI 0.86–1.60[49]).

Six studies were included in the meta-analysis for the outcome “COPD admission” in a comprehensive SFL setting (Fig 4). There was a non significant decrease of 20% after SFL (overall RR = 0.80, 95%CI = 0.63; 1.00). Heterogeneity was high (I2 = 96%, p <0.001). In the sensitivity analysis, the exclusion of individual studies modified the estimates substantially, with pooled RRs of COPD admissions ranging from 0.73 to 0.85. No study was found to be the main origin of heterogeneity between studies, I2 remained above 80% in the sensitivity analysis (S4 Table). Analysis by subgroup showed significant values in the quasi-experimental studies (overall RR = 0.83, 95% CI = 0.74; 0.94, I2 = 40%, p = 0.20), in the low RoB ones (overall RR = 0.84, 95%CI = 0.73; 0.98, I2 = 61%) and in those with less than 24 months follow-up (overall RR = 0.61, 95%CI = 0.59; 0.63; I2 = 0%, p = 0.35) (S6 Table).

### SFL effect on other respiratory diseases admissions

Six papers evaluated: 1) Respiratory diseases taken together (three studies)[36,49,80] and 2) Other respiratory disease admisions (four studies)[48,49,61,75] such as pneumonia and pneumothorax. One paper fell into both categories[49]. Four (66.7%) of the six were in a general population and two exclusively in adults[48,49], five (83.3%) had a non-experimental design, three (50.0%) evaluated comprehensive SFL[48,49,75], and one (16.7%) different types of SFL[36]. The evaluation period ranged from 11 months to three years.

There were discrepant results in overall respiratory admissions (only one paper out of three reported a significant 15.0% decline in adults[49]) and in respiratory infection (two[49,75] of four papers found significant decreases up to 23.0%[49]).

Only two studies could be included in the meta-analysis for lung infections (pneumonia or bronchitis) in adults in a comprehensive SFL setting (Fig 4). There was a non significant decrease of 14% after SFL (overall RR = 0.86, 95% CI = 0.67; 1.10). The between-study heterogeneity was substantial (I2 = 55%, p = 0.13).

### SFL effect on respiratory mortality

Four (8.0%) of the 50 papers were based on mortality data[33,69,70,80]. These studies were heterogeneous with respect to type of population (one study [25.0%] in prisioners[33], two [50.0%] in adults[69,70], and one [25.0%] in a general population[80]), study design (three [75.0%] were non-experimental), type of SFL evaluated (two comprehensive [50.0%] [69,70], one [25.0%] partial[80], and one [25.0%] of different types) and range of post-ban evaluation (from one to seven years).

Two studies (50.0%) performed in Ireland analysed mortality data on respiratory diseases[69,70], and one focused on effects taking into account socioeconomic status[69]. Decreases in mortality rates were primarily due to reductions in passive smoking (these results were supported in that no observable change in smoking prevalence was seen as a result of the SFL), COPD mortality reduction rate was 0.62 (95% CI 0.46–0.83)[70], especially in women (reduction rate 0.47; 95%CI 0.32–0.70)[70] and in the most deprived areas[69]. In North American prisons[33], pulmonary death declined by 29.0% with men having significantly higher rates of death than women for all smoking-related causes. There was only one study about partial SFL which found no significant results[80].

### Quality assessment of included studies

Methodological quality assessment was classified as summary RoB (S2 Table): low in 23 (46.0%) of the 50 papers, moderate in 19 (38.0%), and high in eight (16.0%). In general, the two main weaknesses were selection and attrition bias, and the highest rated domains were detection and reporting bias. The summary RoB was low in 18 (47.37%) of the 38 non-experimental studies and in five (41.67%) out of 12 of the quasi-experimental ones. The most highly rated domains (with a higher proportion of low RoB) in the non-experimental studies were reporting (92.1%) and detection bias 2 (Was the policy unlikely to affect data collection? [89.5%]). The lowest values (with a higher proportion of high RoB) were selection (28.9%), attrittion (18.4%) and other bias (18.4%). For the quasi-experimental studies the most highly rated domains were reporting (100%) and attrition (83.3%), and the worst other bias (16.7%) and confounding bias (16.7%). Papers that evaluated spirometric parameters, respiratory mortality, and sensory symptoms were those that presented the greatest percentages of low RoB (62.5, 50.0, and 47.7%, respectively).

## Discussion

SFL beneficial effects were observed in workers with respect to respiratory and sensory symptomatology. The majority of the studies reported a decrease in hospital admissions for asthma and COPD in all populations (overall population or population stratified by age). Regarding other lung diseases, respiratory mortality, and spirometric parameters, the results are heterogeneous and discrepant. Comprehensive SFL was more commonly evaluated than partial, and periods of assessment ranged from one month to seven years. SFL effect appeared to be greater when the legislation was comprehensive. Due to the reduced number of studies involved in the subgroup analysis, the conclusions of the meta-analysis should be considered with caution. We used a random effect model in order to be able to control heterogeneity. Sensitivity analysis of subgroups showed significant decreases in any respiratory symptoms (both in comprehensive and partial SFL settings) and asthma admissions in comprehensive settings (in adults and children). No significant results were found about the effect of SFL on FEV1, COPD and lung infection admissions. In the rest of the outcomes, either the number of studies involved was very low (FVC, FEF25-75% in comprehensive SFL setting and any sensory symptom in partial SFL setting) or heterogeneity was high despite sensitivity analysis (any sensory symptoms and asthma in a general population in comprehensive SLF settings). All of which hinders extrapolation of data to the whole population, and thus limits the strength of the conclusions drawn.

According to this review, SFL effects are more intense in a worker population with respect to sensory symptoms followed by respiratory ones. In contrast, effects on lung function were not so clear. Spirometry parameters could have been conditioned by other factors such as correct performance of the technique, whether the participant was a smoker or asthmatic and, in the case of the latter, whether asthma medication was being taken[87]. In fact, in one of the included studies the authors were unable to analyze the values due to the difficulties in gathering data (participants not fully co-operating in the test)[32]. Overall, asthma and COPD were the diseases most assessed. The majority of the studies reported favourable results for SFL with a maximum decrease in hospital admissions for adults of 46.0%[48] and 36.0%[49] for COPD and asthma, respectively. Few studies have evaluated SFL impact on other respiratory diseases and mortality, and their results have been heterogeneous. Nevertheless, an immediate 38.0% decrease in mortality due to COPD has been reported[70].

Most of the included studies analysed the impact of comprehensive SFL which can cause the greatest decrease in sensory and respiratory symptoms particularly in the immediate post-ban period. It is possible that, at long-term, these effects in worker population are not perceived due to the situation becoming normalized, that is to say, without exposure to second-hand smoke due to SFL. In the studies carried out in all populations, effects were evaluated at eleven months after SFL implementation and better results were observed with respect to decreased admissions due to respiratory diseases in locations with the strictest SFL. Nevertheless, data on respiratory mortality are scarce and from heterogeneous populations. In addition, the maximum time period for SFL implementation to be studied is 6.75 years[69] and differences have not yet been found between the time period immediately after SFL and long-term[70].

Some studies have performed analysis in subgroups and reported a greater decrease in the percentage of sensory and respiratory symptoms in a working, non-smoking population[43,52,76], greater decrease in COPD mortality in women[33,69,70] and in people older than 65 years[69,70], and fewer asthma admissions in a population aged over 20 years[64] and between 30–39 years[49]. Most studies detected a decrease of less intensity in children than in adults for asthma admissions after SFL implementation. However, the few studies that compared both populations reported heterogeneous findings. One explanation is that adults may be exposed to secondhand smoke at both home and work whilst children are only exposed at home[14,64].

In contrast with previous reviews that provided data on respiratory symptomatology and admissions, we identified a considerably larger number of studies (50 papers compared to 21 in the most extensive review by other authors)[21]. The review by Polanska *et al*[87] was limited to respiratory and sensory symptoms in a worker population. They found a reduction in respiratory symptoms in ten out of 12 studies, and in all except one of these ten studies a decline in sensory symptoms. Callinan *et al*[8] arrived at similar conclusions in their systematic review and mentioned, furthermore, a clear reduction in sensory symptoms in smokers and non-smokers. In that review they analyzed spirometer parameters from five studies: in two there were significant increases in FEV1; in three significant increases in FVC; and in three significant reductions in FEF. With respect to symptomatology and lung function the results mentioned concur with those we obtained in our systematic review: 26 papers were identified related to respiratory symptoms, 19 to sensory symptoms, and eight to lung function. In contrast, the 2010 Cochrane[8] identified 12, ten, and six papers, respectively. Self-reported symptoms were excluded from the 2016 up-dated Cochrane Review[21] and symptomatology evidence was not updated with respect to the 2010 edition. In contrast, our review found 12 papers more in the period 2009–2015. The 2016 Cochrane Review selected papers that evaluated effects on health when follow-up was a minimum of six months post-ban (no restriction in our review) and institutional settings were not included. It was concluded that data regarding asthma and COPD admissions were inconsistent. Tan and Glantz[23], in their meta-analysis that included eight articles about respiratory disease admissions with a heterogeneity of 88%, obtained a relative risk of 0.760 (95% CI 0.682–0.846) for decreased asthma and lung infections without any statistically significant association for COPD and spontaneous pneumothorax (fewer studies), and without any follow-up time differences. According to our data, asthma in the general population decreased by 13% and asthma in adults by 23%, with no significant findings for COPD and lung infection. Been *et al*[88] in their meta-analysis based on three studies about asthma admission in children (with moderate bias risk) obtained an overall reduction of 10.10% (95% CI -15.2 to -5), and a non-significant trend towards an annual decrease rate (-7.5% per year, 95% CI -16 to 0.9). In this meta-analysis, a significant overall RR of 0.82 was found. According to the narrative synthesis, we observed that most articles reported a decrease in the number of hospital/non-hospital emergency admissions due to asthma (75.6% of papers) and/or COPD (66.7% of papers). However, as some of these papers only found evidence in population subgroups (children or adults), the results are inconclusive. With respect to the meta-analysis, it seems that SFL has a protective effect nevertheless due to the high heterogeneity between studies of the outcomes analysed the conclusions should be drawn with caution.

Most of the studies in this review had a non-experimental design. The lack of a control group only permits the evaluation of variations before and after SFL implementation; it does not allow the overall observed changes to be attributed to the legislation implemented. We have observed that in some studies significant results were only obtained when comparing locations with and without SFL or time periods with varying restrictions with respect to smoking bans[61].

The fact that the data source of the included studies with respect to symptomatology (self-administered International Union Against Tuberculosis and Lung Disease questionnaires) and hospital admissions (central registers) has been quite homogenous is a strength as it permits comparison with findings amongst different studies. However, self-reported data have limited validation compared to objective measures. There was a lack of simultaneous adjustment and stratification for confounders in some studies. A few of them stratified confounders such as sex[31,35], location[35,37,77], socioeconomic status[35,59,69], seasonality[64], smoking status[52], secondhand smoke[47,77], influenza/air polution[47], number of symptoms[31,63], comprehensiveness of SFL[54], population growth[60], different policies on tobacco[36], and being resident or not[48,60]. SFL is not the only measure that has had an impact on passive smoking and tobacco-related disease. Other actions that have influenced the consumption of tobacco are control of publicity, tax increases resulting in higher price[89,90], and restrictions on the sale of tobacco products[8].Very often these steps are introduced at the same time as the new legislation comes into being. A policy may be considered ineffective when other components simultaneously occur which cause its impact to be under-rated. It might, therefore, be difficult to know how these different actions could influence the impact observed. As a result, it is important to perform sensitive analysis by subgroups[24].

In general, most articles were classified as low or moderate RoB with the non-experimental ones having a slightly higher percentage. Selection and attrition biases had the worst results, very probably due to convenience sampling (in the case of selection bias) in the studies on symptomatology. Moreover, losses of participants in the hospitality sector are habitual due to the temporal nature of this kind of employment[32,91]. In the twelve quasi-experimental studies (the majority about respiratory diseases in the general population) the confounding bias domain was the worst evaluated. This shows that in even the most robustly designed study it is necessary to analyze confounding factors that could over or underestimate SFL effect. In the systematic review of Frazer *et al*[21], a different assessment tool was used: “adequate sequence generation”, “adequate allocation concealments”, and “blinding of personnel/all outcomes” (more suitable for randomized clinical trials). These domains were adapted in this systematic review due to the type of studies (evaluating a policy). Papers were rated better in this systematic review than in the up-dated Cochrane Review. However, quality assessment of each paper were performed by two authors independently and checked by a third in the case of discrepancies.

### Strengths and limitations

To the best of our knowledge, this is the first scientific review performed with respiratory outcomes (symptoms, functionality, and hospital admissions) as an effect of SFL, measured in all groups (workers, adults, children, and general populations). The rigorous procedures employed (paired reviewers and a third one in the case of discrepancies) have ensured the validity of the data extraction. A detailed synthesis of sensory and respiratory symptomatology has been done (excluded from the up-dated Cochrane Review). Grey literature was not employed but the combination of heterogeneous sources of data adds value to the results. We tried to identify all the possible papers appearing in scientific journals from six different databases in addition to manually searching the references in the papers and consulting experts. One of the inclusion criteria was that papers were written in English, French, Portuguese, Italian, Catalan or Spanish. This did not have an effect on our results as only one document had to be excluded (Norway)[92].

Although identification and selection biases are common threats to validity in all systematic reviews, they are more likely in the case of non-randomized studies where registration is not standard practice[93]. Particular effort has been devoted to reducing identification bias, as shown by our search strategy which included several databases.

Variability in participants, type of SFL, outcome measures and definition, duration of follow-up and study design may affect the impact of SFL. In addition, the considerable heterogeneity observed hinders the drawing of conclusions about SFL from the meta-analysis. However, the narrative synthesis helps to investigate similarities and differences among studies as well to explore any patterns in the data to better understand the impact of SFL. A possible bias due to authors who may have had a financial conflict of interest arising from the tobacco industry is a complex issue. In fact, with respect to studies on passive smoking there are a number of such authors. Nevertheless, this type of article generally has low quality scores and was probably excluded from selection[94].

### Future lines of study

Building upon the present observations, we would like to underline the need to address several important issues for future research. None of the studies reported sensory/respiratory symptoms and lung function in the general population. Moreover, the effect of SFL with respect to respiratory symptomatology in children was only evaluated in two studies without conclusive results. It is notable that non-hospital setting was monitored in only two studies[34,79]. Neither are there data about respiratory mortality in worker and child populations. There are few, heterogeneous studies regarding lung function, medication use, respiratory infections (both upper and lower tracts), and mortality. In addition, it is essential to better study the mid- to long-term effects of SFL on mortality, COPD, and another chronic respiratory diseases. Finally, we believe there is a need for quasi-experimental design studies comparing locations with and without SFL, and partial versus comprehensive SFL, to better confirm its effects. Moreover, we consider that analysis by age, gender, and socio-economic factors are necessary given that tobacco consumption may vary.

## Conclusions

Results appear to indicate that comprehensive SFL decreases sensory symptomatology more than partial. Almost all the studies reported effectiveness of SFL in respiratory and sensory symptoms in workers and children with significance that decreased in the meta-analysis. There is a majority of studies denoting the effectiveness of SFL in admissions for asthma and COPD in all populations but without statistical significance for the latter in the meta-analysis. There are, however, few studies about respiratory mortality, respiratory infection, and lung function and they do not demonstrate strong effectiveness. It can be concluded, therefore, that it is important to continue conducting research into SFL effectiveness particularly in areas lacking results that can contribute to the available evidence.

## Supporting information

S1 PRISMA checklist (DOC)

S1 Table Search strategy applied in the different databases: SFL effects on respiratory and sensory disorders. (PDF)

S2 Table Quality assessment of the risk of bias of papers evaluating SFL effects on respiratory and sensory disorders (1995–2015). (PDF)

S3 Table Descriptive characteristics of the 50 articles obtained for smokefree legislation effects on respiratory disorders (1995–2015). (PDF)

S4 Table Sensitivity analysis by omitting one or two until I2 dropped below the intented threshold 50% and range. (PDF)

S5 Table Subgroup analysis by study design and risk of bias among studies relating any respiratory and sensory symptoms. (PDF)

S6 Table Subgroup analysis by study design and risk of bias among studies relating asthma and COPD admissions in comprehensive SFL setting. (PDF)

S1 Fig Funnel plot of any respiratory symptom in comprehensive smokefree legislation setting. Publication bias.
(PDF)
