# Supplementary material for: Smokefree legislation effects on respiratory and sensory disorders: A systematic review and meta-analysis
Source: PLoS One. 2017 Jul 31;12(7):e0181035. doi: 10.1371/journal.pone.0181035 (PMC5536320; doi:10.1371/journal.pone.0181035)
Supplement: S1 Table — (PDF) [file pone.0181035.s002.pdf]

Table S1: Search strategy applied in the different databases: SFL effects on respiratory and sensory disorders

Web of Science	Scopus
1.("Chronic Obstructive Pulmonary Disease" OR "COPD" OR "Chronic Obstructive Lung Disease"OR "Common Cold" OR Bronchitis OR "spontaneous pneumothorax" OR Pneumonia OR otitis OR Nasopharyngitis OR sinusitis OR "Pulmonary Disease, Chronic Obstructive" OR "Common Cold" OR "Bronchitis, Chronic" OR "Bronchitis" OR "Pneumothorax" OR "Pneumonia" OR "Otitis" OR "Nasopharyngitis" OR "Sinusitis" OR "Asthma" OR asthma OR "Respiratory Tract Diseases" OR "Respiration Disorders" OR respiratory)	1. TITLE-ABS-KEY ("Chronic Obstructive Pulmonary Disease" OR "COPD" OR "Chronic Obstructive Lung Disease" OR "Common Cold" OR bronchitis OR "spontaneous pneumothorax" OR pneumonia OR otitis OR nasopharyngitis OR sinusitis OR "Pulmonary Disease, Chronic Obstructive" OR "Common Cold" OR "Bronchitis, Chronic" OR "Bronchitis" OR "Pneumothorax" OR "Pneumonia" OR "Otitis" OR "Nasopharyn gitis" OR "Sinusitis" OR "Asthma" OR asthma OR "Respiratory Tract Diseases" OR "Respiration Disorders" OR respiratory)<br>AND SUBJAREA (mult OR medi OR nurs OR vete OR dent OR heal OR envi). Health sciences: Medicine, nursing, veterinary, dentisty, health professions, multidisciplinary. Physical sciences: environement sciences<br>AND PUBYEAR > 1994 hasta 21/2/2015<br>Results: 562.765 records
2.("smoking ban" OR "smoking bans" OR "smokefree law" OR "smokefree laws" OR "smoke-free law" OR "smoke-free laws" OR "smoke-free policy" OR "smoke-free policies" OR "smokefree policy" OR "smokefree policies" OR "Smoke-Free Policy" OR "smoke-free bans" OR "smoke-free ban" OR "smokefree bans" OR "smokefree ban" OR "smoking bans" OR "smoking ban" OR "smoking legislation" OR "legislative smoking" OR "tobacco legislation" OR "tobacco legislations" OR "tobacco laws" OR "smoke-free legislation" OR "smoke-free legislations" OR "smokefree legislation" OR "smokefree legislations" OR "smoking policies" OR "smoking policy" OR anti-tobacco policy OR "Smoking/legislation and jurisprudence" OR "Tobacco Smoke Pollution/legislation and jurisprudence") NOT ("Electronic cigarettes" OR "motivational interviewing" OR "randomized clinical trial" OR Randomized Controlled Trial OR qualitative study OR oxidat* OR economic OR genetic OR motivational)	2. TITLE-ABS-KEY ("smoking ban" OR "smoking bans" OR "smokefree law" OR "smokefree laws" OR "smoke-free law" OR "smoke-free laws" OR "smoke-free policy" OR "smoke-free policies" OR "smokefree policy" OR "smokefree policies" OR "Smoke-Free Policy" OR "smoke-free bans" OR "smoke-free ban" OR "smokefree bans" OR "smokefree ban" OR "smoking bans" OR "smoking ban" OR "smoking legislation" OR "legislative smoking" OR "tobacco legislation" OR "tobacco legislations" OR "tobacco laws" OR "smoke-free legislation" OR "smoke-free legislations" OR "smokefree legislation" OR "smokefree legislations" OR "smoking policies" OR "smoking policy" OR anti-tobacco policy OR "Smoking/legislation and jurisprudence" OR "Tobacco Smoke Pollution/legislation and jurisprudence")<br>AND SUBJAREA (mult OR medi OR nurs OR vete OR dent OR heal OR envi). Health sciences: Medicine, nursing, veterinary, dentisty, health professions, multidisciplinary. Physical sciences: environement sciences<br>AND PUBYEAR > 1994 until 21/2/2015<br>Results: 1698 records
3. #1 AND #2	3. #1 AND #2
4. Limit from 1995 to 2015	
Results: 258 records	Results: 699 records

Cochrane Library	Google Scholar
Keywords; smoking ban	With all the words: Smoking ban<br>With at least one of the words: law smokefree asthma respiratory<br>Without the words: randomized “electronic cigarette” myocardial<br>Show articles dated between1995-2015<br>Results: 16900 records.
Publication Year from 1995 to 2015, in Cochrane Reviews (Reviews and Protocols) and Other Reviews	A preliminary Google Scholar search showed a large number of hits, so we scanned the first 200 results and we chose 28 for inclusion criteria.
Excluding papers from Trials (44) and Economic Evaluations (1)	
Results: 5 records	Results: 28 records after reading title of 200 first searches
