# Supplementary material for: Smokefree legislation effects on respiratory and sensory disorders: A systematic review and meta-analysis
Source: PLoS One. 2017 Jul 31;12(7):e0181035. doi: 10.1371/journal.pone.0181035 (PMC5536320; doi:10.1371/journal.pone.0181035)
Supplement: S2 Table — (PDF) [file pone.0181035.s003.pdf]

Table S2. Quality assessment of the risk of bias of papers evaluating SFL effects on respiratory and sensory disorders (1995-2015)

Risk of bias was evaluated through the following items: confounding bias, detection bias (only in non-experimental studies- two domains), selection bias, attrition bias, reporting bias, other bias.

- Confounding bias:
    - Quasi-experimental studies: Comparability of groups.
    - Non-experimental studies: Was the policy independent of other changes?
- Detection bias:
    1. Was the shape of the policy effect pre-specified?
    2. Was the policy unlikely to affect data collection?
- Selection bias: Sample representativeness.
- Attrition bias: Describe the completeness of outcome data for each main outcome, including attrition and exclusions from the analysis.
- Reporting bias: State how the possibility of selective outcome reporting was examined by the review authors, and what was found.
- Other bias: State any important concerns about bias not addressed in the other domains in the tool.

We rated the overall methodological quality of the included studies as being at low, moderate, or high risk of bias (RoB). Depending on the type of design:

- Non experimental studies: without control group and seven domains of bias. The articles where there are 5 or more subcategories with low bias are classified as "Low" in the summary risk of bias; the ones with a number of subcategories between 3 and 4 classified as Low are rated as "Moderate"; and the ones with a number of subcategories between 1 and 2 classified as "Low" are rated as "High".
- Quasi-experimental studies: with control group and five domains of bias. The articles where there are 4 or more subcategories with low bias are classified as "Low" in the summary risk of bias; the ones with a 3 subcategories classified as Low are rated as "Moderate"; and the ones with a number of subcategories between 1 and 2 classified as "Low" are rated as "High".

**Non experimental studies**								
**Study, year**	**Confounding bias**	**Detection bias 1**	**Detection bias 2**	**Selection bias**	**Attrition bias**	**Reporting bias**	**Other bias**	**Summary risk of bias**
Ayres, 2009[1]	Low	Low	Low	Unclear	Low	Low	Low	Low
Bannon, 2009[2]	Low	Low	Low	High	Unclear	Low	Low	Low
Croghan, 2015[3]	Low	Low	Low	Low	Unclear	Low	Unclear	Low
Dilley, 2012[4]	Unclear	Low	Low	Low	Unclear	Low	Unclear	Low
Durham, 2011[5]	Low	Low	Low	High	High	Low	High	Low
Eagan, 2006[6]	Low	Low	Low	Low	Unclear	Low	Unclear	Low
Eisner, 1988[7]	Unclear	Low	Low	Unclear	Low	Low	Low	Low

Non experimental studies (continuation)								
**Study, year**	**Confounding bias**	**Detection bias 1**	**Detection bias 2**	**Selection bias**	**Attrition bias**	**Reporting bias**	**Other bias**	**Summary risk of bias**
Farrelly, 2015[8]	Low	Unclear	Low	High	High	Low	Low	Moderate
Goodman, 2007[9]	Low	Low	Low	High	Low	Low	Low	Low
Hahn, 2006[10]	Low	Low	Low	Low	High	Low	High	Low
Ho, 2010[11]	Unclear	Low	Unclear	Unclear	Unclear	Low	Unclear	High
Humair, 2014[12]	Unclear	Low	Low	Unclear	Low	Low	High	Moderate
Kalkhoran, 2015[13]	Low	Unclear	Low	Low	Low	Low	High	Low
Kent, 2012[14]	Unclear	Low	Unclear	Low	Low	Low	Unclear	Moderate
Kim, 2014[15]	High	High	Low	High	Unclear	Low	Unclear	High
Larsson, 2008[16]	Unclear	Low	Low	High	High	Low	High	Moderate
Li, 2013[17]	High	Low	Low	High	Low	Low	High	Moderate
MacCalman, 2012[18]	Unclear	Unclear	Unclear	High	High	Low	Unclear	High
Mackay, 2010[19]	Low	Low	Low	Low	Unclear	Low	Unclear	Low
Madureira,2012[20]	Unclear	Low	Low	High	High	Low	Unclear	Moderate
Madureira,2014[21]	Unclear	Low	Low	High	High	Low	Unclear	Moderate
McGhee, 2014[22]	Low	Unclear	Low	Low	Unclear	Low	Unclear	Moderate
Menzies, 2006[23]	Unclear	Low	Low	Low	Unclear	Unclear	Low	Moderate
Millet, 2013[24]	Low	Unclear	Low	Low	Low	Low	Low	Low
Pearson, 2009[25]	Unclear	Unclear	Low	Unclear	Unclear	Low	Unclear	High
Rajkumar, 2014[26]	Low	Low	Low	Low	Unclear	Low	Unclear	Low
Rayens, 2008[27]	Low	Unclear	Low	Low	Unclear	Low	Unclear	Moderate
Reijula, 2012[28]	Low	Low	Low	Unclear	Unclear	Low	Low	Low
Roberts, 2012[29]	Unclear	Low	Low	Unclear	Low	Low	Unclear	Moderate
Schoj, 2010[30]	Low	Low	Low	Unclear	Low	Low	Unclear	Low
Sims, 2013[31]	Unclear	Low	Low	Low	Low	Unclear	High	Moderate
Skogstad, 2006[32]	High	Low	Low	Unclear	Unclear	Low	Low	Moderate
Stallings-Smith, 2013[33]	Low	Low	Low	Low	Low	Low	Unclear	Low
Stallings-Smith, 2014[34]	Low	Low	Low	Low	Low	Low	Low	Low
Vinnikov, 2013[35]	Low	Low	Low	Unclear	Low	Low	Unclear	Low
Wieslander, 2000[36]	Unclear	Low	High	Low	Unclear	Unclear	Unclear	High
Wilson, 2012[37]	Unclear	Low	Low	High	Low	Low	Unclear	Moderate
Yildiz, 2014[38]	Unclear	Unclear	Low	Low	Unclear	Low	Unclear	Moderate

Quasi-experimental studies								
**Study, year**	**Confounding bias**	**Detection bias 1**	**Detection bias 2**	**Selection bias**	**Attrition bias**	**Reporting bias**	**Other bias**	**Summary risk of bias**
Allwright, 2005[39]	Low	Not applicable	Not applicable	Unclear	Low	Low	Low	Low
Binswanger,2014[40]	Low	Not applicable	Not applicable	Low	Unclear	Low	High	Moderate
Dove, 2012[41]	Unclear	Not applicable	Not applicable	Low	Unclear	Low	Unclear	High
Dusemund, 2013[42]	Low	Not applicable	Not applicable	Low	Low	Low	Unclear	Low
Fernández, 2009[43]	Low	Not applicable	Not applicable	Unclear	Low	Low	Unclear	Moderate
Gaudreau, 2013[44]	Low	Not applicable	Not applicable	Low	Low	Low	Unclear	Low
Head, 2012[45]	High	Not applicable	Not applicable	Unclear	Low	Low	Unclear	High
Herman, 2011[46]	Unclear	Not applicable	Not applicable	Low	Low	Low	Unclear	Moderate
Landers, 2014[47]	Low	Not applicable	Not applicable	Low	Low	Low	Unclear	Low
Moraros, 2010[48]	High	Not applicable	Not applicable	Low	Low	Low	Low	Low
Naiman, 2010[49]	Unclear	Not applicable	Not applicable	Low	Low	Low	Unclear	Moderate
Vander, 2012[50]	Unclear	Not applicable	Not applicable	Unclear	Low	Low	High	High

REFERENCES

1. Ayres JG, Semple S, Maccalman L, Dempsey S, Hilton S, Hurley JF, et al. (2009) Bar workers' health and environmental tobacco smoke exposure (BHETSE): symptomatic improvement in bar staff following smoke-free legislation in Scotland. Occup Environ Med 66:339–46
2. Bannon F, Devlin A, McElwee G, Gavin A (2009) Greater gains from smoke- free legislation for non-smoking bar staff in Belfast. Eur J Public Health 087
3. Croghan IT, Ebbert JO, Hays JT, Schroeder DR, Chamberlain AM, Roger VL, et al. (2015) Impact of a countywide smoke-free workplace law on emergency department visits for respiratory diseases: a retrospective cohort study. BMC Pulm Med 15:6.
4. Dilley JA, Harris JR, Boysun MJ, Reid TR (2012) Program, policy, and price interventions for tobacco control: Quantifying the return on investment of a state tobacco control program. Am J Public Health 102:e22–28.
5. Durham AD, Bergier S, Morisod X, Locatelli I, Zellweger JP, et al. (2011) Improved health of hospitality workers after a Swiss cantonal smoking ban. Swiss Med Wkly 141: w13317 doi:10.4414/smw.2011.13317.
6. Eagan TML, Hetland J, Aarø LE (2006) Decline in respiratory symptoms in service workers five months after a public smoking ban. Tob Control 15(3):242– 246
7. Eisner MD, Smith AK, Blanc PD (1998) Bartenders' respiratory health after establishment of smoke-free bars and taverns. JAMA 280:1909–1914.
8. Farrelly MC, Nonnemaker JM, Chou R, et al. (2005) Changes in hospitality workers' exposure to secondhand smoke following the implementation of New York's smoke-free law. Tob control 14:236–241.
9. Goodman P, Agnew M, McCaffrey M, Paul G, Clancy L (2007) Effects of the Irish smoking ban on respiratory health of bar workers and air quality in Dublin pubs. Am J Respir Crit Care Med 175:840–845.
10. Hahn EJ, Rayens MK, York N, Okoli CTC, Zhang M, Dignan M, et al. (2006). Effects of a smoke-free law on hair nicotine and respiratory symptoms of restaurant and bar workers. J Occup Environ Med 48:906–13.
11. Ho S, Wang M, Lo W, Mak K (2010) Comprehensive smoke-free legislation and displacement of smoking into the homes of young children in Hong Kong. Tob control 19:129-33.
12. Humair J-P, Garin N, Gerstel E, Carballo S, Carballo D, Keller P-F, et al. (2014) Acute Respiratory and Cardiovascular Admissions after a Public Smoking Ban in Geneva, Switzerland. PLoS ONE 9(3): e90417. doi:10.1371/journal.pone.0090417.
13. Kalkhoran S, Sebrie EM, Sandoya E, Glantz SA (2015) Effect of Uruguay's National 100% Smokefree Law on Emergency Visits for Bronchospasm. Am J Prev Med 49:85–88.
14. Kent BD, Sulaiman I, Nicholson TT, Lane SJ, Moloney ED (2012) Acute pulmonary admissions following implementation of a national workplace smoking ban. Chest 142: 673–679.
15. Kim J, Kwon H-J, Lee K, Lee D-H, Paek Y, Kim S-S, et al. (2015) Air Quality, Biomarker Levels, and Health Effects on Staff in Korean Restaurants and Pubs Before and After a Smoking Ban. Nicotine Tob Res
16. Larsson M, Boëthius G (2008) Exposure to environmental tobacco smoke and health effects among hospitality workers in Sweden—before and after the implementation of a smoke-free law. Scan J Work Environ Health 267-277.
17. Li X, Gao J, Zhang Z, Wei M, Zheng P, Nehl EJ, et al. (2013) Lessons from an Evaluation of a Provincial-Level Smoking Control Policy in Shanghai, China. PLoS ONE 8(9): e74306. doi:10.1371/journal.pone.0074306.
18. MacCalman L, Semple S, Galea KS, Van Tongeren M, Dempsey S, Hilton S, et al. (2012) The relationship between workers self-reported changes in health and their attitudes towards a workplace intervention: Lessons from smoke-free legislation across the UK hospitality industry. BMC Public Health 12.
19. Mackay D, Haw S, Ayres JG, Fischbacher C, Pell JP (2010) Smoke-free Legislation and Hospitalizations for Childhood Asthma. N Engl J Med 363: 1139–1145.
20. Madureira J, Mendes A, Almeida S, Teixeira JP (2012) Positive impact of the Portuguese smoking law on respiratory health of restaurant workers. J Toxicol Environ Heal - Part A Curr Issues 75:776–787.
21. Madureira J, Mendes A, Teixeira Paulo J (2014) Evaluation of a Smoke-Free Law on Indoor Air Quality and on Workers' Health in Portuguese Restaurants. J Occup Environ Hyg 11:201–209.

22. McGhee SM, Wong CM, Schooling CM, Thomas GN, Hedley AJ, Chau J, et al. (2014) Smoke-free policies on population health outcomes. Hong Kong Med J 20(Suppl 3):36–41.
23. Menzies D, Nair A, Williamson PA, Schembri S, Al-Khairalla MZH, Barnes M, et al. (2006) Respiratory symptoms, pulmonary function, and markers of inflammation among bar workers before and after a legislative ban on smoking in public places. JAMA 296: 1742–1748.
24. Millett C, Lee JT, Laverty AA, Glantz SA, Majeed A (2013) Hospital admissions for childhood asthma after smoke-free legislation in England. Pediatrics 131: e495–501.
25. Pearson J, Windsor R, El-Mohandes A, Perry DC (2009) Evaluation of the immediate impact of the Washington, D.C., smoke-free indoor air policy on bar employee environmental tobacco smoke exposure. Public Health Rep 124 Suppl:134–142.
26. Rajkumar S, Stolz D, Hammer J (2014) Effect of a Smoking Ban on Respiratory Health in Nonsmoking Hospitality Workers: A Prospective Cohort Study. JOEM 56: e86-e91.
27. Rayens MK, Burkhart P V, Zhang M, Lee S, Moser DK, Mannino D, et al. (2008) Reduction in asthma-related emergency department visits after implementation of a smoke-free law. J Allergy Clin Immunol 22: 537–541.
28. Reijula JP, Johnsson TS-E, Kaleva PS, Reijula KE (2012) Exposure to tobacco smoke and prevalence of symptoms decreased among Finnish Restaurant workers after the smoke-free law. Am J Ind Med 55:37–43.
29. Roberts C, Davis PJ, Taylor KE, Pearlman DN (2012) The impact of Rhode Island’s statewide smoke-free ordinance on hospital admissions and costs for acute myocardial infarction and asthma. Med Health R I 95:23–25.
30. Schoj V, Alderete M, Ruiz E, Hasdeu S, Linetzky B, Ferrante D (2010) The impact of a 100% smoke-free law on the health of hospitality workers from the city of Neuquén, Argentina. Tob Control 19:134–137
31. Sims M, Maxwell R, Bauld L, Gilmore A (2010) Short term impact of smoke- free legislation in England: retrospective analysis of hospital admissions for myocardial infarction. BMJ 340: c2161 doi:10.1136/bmj.c2161.
32. Skogstad M, Kjærheim K (2006) Cross shift changes in lung function among bar and restaurant workers before and after implementation of a smoking ban. Occup Environ Med 63:482-487
33. Stallings-Smith S, Zeka A, Goodman P, Kabir Z, Clancy L (2013) Reductions in cardiovascular, cerebrovascular, and respiratory mortality following the national Irish smoking ban: interrupted time-series analysis. PLoS One 8: e62063.
34. Stallings-Smith S, Goodman P, Kabir Z, Clancy L, Zeka A (2014) Socioeconomic Differentials in the Immediate Mortality Effects of the National Irish Smoking Ban. PLoS ONE 9(6): e98617. doi:10.1371/journal.pone.0098617
35. Vinnikov D, Blanc PD, Brimkulov N, Redding-Jones R (2013) Five-year lung function observations and associations with a smoking ban among healthy miners at high altitude (4000 m). J Occup Environ Med 55:1421–1425.
36. Wieslander G, Lindgren T, Norback D, Venge P. (2000). Changes in the ocular and nasal signs and symptoms of aircrews in relation to the ban on smoking an intercontinental flights. Scand J Work Environ Heal 26:514–22.
37. Wilson T, Shamo F, Boynton K, Kiley J (2012) The impact of Michigan’s Dr Ron Davis smoke-free air law on levels of cotinine, tobacco-specific lung carcinogen and severity of self-reported respiratory symptoms among non-smoking bar employees. Tob Control 21:593–595.
38. Yildiz F, Baris SA, Basyigit I, Boyaci H, Aydinlik H, Sonmez PO (2014) Role of smoke-free legislation on emergency department admissions for smoking-related diseases in Kocaeli, Turkey. East Mediterr Health J 20:774–780. .
39. Allwright S, Paul G, Greiner B, Mullally BJ, Pursell L, Kelly A, et al. (2005) Legislation for smoke-free workplaces and health of bar workers in Ireland: before and after study. BMJ 331:1117–1120.
40. Binswanger IA, Carson EA, Krueger PM, Mueller SR, Steiner JF, Sabol WJ (2014) Prison tobacco control policies and deaths from smoking in United States prisons: Population based retrospective analysis. BMJ 349
41. Dove MS, Dockery DW, Connolly GN (2011) Smoke-free air laws and asthma prevalence, symptoms, and severity among nonsmoking youth. Pediatrics 127:102–109
42. Dusemund F, Baty F, Brutsche MH (2014) Significant reduction of AECOPD hospitalisations after implementation of a public smoking ban in Graubünden, Switzerland. Tob Control;tobaccocontrol--2013.
43. Fernandez E, Fu M, Pascual JA, Lopez MJ, PerezRios M et al. (2009) Impact of the Spanish smoking law on exposure to second-hand smoke and respiratory health in hospitality workers: a cohort study. PLOS ONE 4: e4244. doi:10.1371/journal.pone.0004244.
44. Gaudreau K, Sanford CJ, Cheverie C, McClure C (2013) The Effect of a Smoking Ban on Hospitalization Rates for Cardiovascular and Respiratory Conditions in Prince Edward Island, Canada. PLoS ONE 8: e56102.
45. Head P, Jackson E B, Bae S, Cherry D et al. (2012) Hospital Discharge Rates Before and After Implementation of a City-wide Smoking Ban in a Texas City, 2004-2008. Prev Chronic Dis 9:E179–E179.
46. Herman PM, Walsh ME (2010) Hospital Admissions for Acute Myocardial Infarction, Angina, Stroke, and Asthma After Implementation of Arizona's Comprehensive Statewide Smoking Ban. Am J Public Health doi:10.2105/AJPH.2009.179572.
47. Landers G. The impact of smoke-free laws on asthma discharges: a multistate analysis (2014) Am J Public Health 104:e74-79.
48. Moraros J, Bird Y, Chen S, Buckingham R, Meltzer RS, Prapasiri S, et al. (2010) The impact of the 2002 delaware smoking ordinance on heart attack and asthma. Int J Environ Res Public Health 7:4169–4178.
49. Naiman A, Glazier RH, Moineddin R (2010) Association of anti-smoking legislation with rates of hospital admission for cardiovascular and respiratory conditions. CMAJ 182: 761–767.
50. Vander Weg MW, Rosenthal GE, Vaughan Sarrazin M (2012) Smoking bans linked to lower hospitalizations for heart attacks and lung disease among medicare beneficiaries. Health Aff 31:2699–2707.
