# Supplementary material for: Smokefree legislation effects on respiratory and sensory disorders: A systematic review and meta-analysis
Source: PLoS One. 2017 Jul 31;12(7):e0181035. doi: 10.1371/journal.pone.0181035 (PMC5536320; doi:10.1371/journal.pone.0181035)
Supplement: S3 Table — (PDF) [file pone.0181035.s004.pdf]

Table S3: Descriptive characteristics of the 50 articles obtained for smokefree legislation effects on respiratory disorders (1995-2015)

Variable	Category	N	% of the papers included
Continent	Europe	24	48
	America	21	42
	Asia	5	10
Country	United States	16	32
	Ireland	6	12
	Switzerland	4	8
	China	3	6
	Scotland	3	6
	England	2	4
	Canada	2	4
	Norway	2	4
	Portugal	2	4
	Sweden	2	4
	Argentina	1	2
	Finland	1	2
	Korea Rep	1	2
	Kyrgyzstan	1	2
	Scotland & England	1	2
	Spain	1	2
	Turkey	1	2
	Uruguay	1	2
Setting	Hospitality	22	44
	City/region	22	44
	Different workplaces	3	6
	Schools	2	4
	Prisons	1	2
Type of legislation*	Total comprehensive	33	66
	Partial comprehensive	12	24
	Different types of SFL	3	6
	Labor	2	4
Study design	Before-after non-experimental design	27	54
	Non-experimental design: time series	10	20
	Quasi-experimental design: time series	6	12
	Before-after quasi-experimental design	5	10
	Other designs	2	4
Participants	Specifically adult workers	25	50
	Adults + children together (general population)	12	24
	Adults	8	16
	Children	4	8
	Vulnerable population (prisoners)	1	2
Data source/data information	Hospital registers	19	38
	Self-reported questionnaires	15	30
	No self-reported questionnaire	4	8
	Self-reported questionnaires + Spirometry	4	8
	Mortality data	3	6
	Spirometry	2	4
	No self-reported questionnaire + Spirometry	2	4
	Hospital registers + Mortality data	1	2

Variable	Category	N	% of the papers included
Study period before the SFL	0-1 month	11	22
	2-6 months	8	16
	7-12 months	1	2
	>1-2 years	7	14
	>2-3 years	4	8
	>3 years	13	26
	Not specified	6	12
Study period after the SFL	0-1 month	4	8
	2-6 months	6	12
	7-12 months	13	26
	>1-2 years	11	22
	>2-3 years	4	8
	>3 years	10	20
	Not specified	2	4
Respiratory and sensory outcomes †	Respiratory symptoms	26	52
	Sensory symptoms	19	38
	Hospital admissions for asthma	17	34
	Hospital admissions for COPD	9	18
	Spirometric parameters	8	16
	Hospital admissions for other pulmonary diseases	4	8
	Other mortality respiratory diseases	3	6
	Hospital admissions for overall respiratory diseases	3	6
	COPD mortality	2	4
	Persistent ear infection	1	2
Declared Conflict of Interest	Yes	31	62
	No	19	38
Summary Risk of Bias	Low	23	46
	Moderate	19	38
	High	8	16

* We classified comprehensive smoke-free legislations as those that banned smoking in virtually all indoor workplaces and public areas, including bars, restaurants and public transport; and parcial as that which comprises fewer locations than those described[6]

All the percentatges of variables add up to100% except: † There are papers with more than one outcome.

COPD=chronic obstructive pulmonary disease.

Reference

6. Hyland A, Barnoya J, Corral JE (2012) Smoke-free air policies: past, present and future. Tob Control 21:154–161.
