# Supplementary material for: Smokefree legislation effects on respiratory and sensory disorders: A systematic review and meta-analysis
Source: PLoS One. 2017 Jul 31;12(7):e0181035. doi: 10.1371/journal.pone.0181035 (PMC5536320; doi:10.1371/journal.pone.0181035)
Supplement: S4 Table — (PDF) [file pone.0181035.s005.pdf]

S4 Table. Sensitivity analysis by omitting one or two until I2 dropped below the intented threshold 50% and range.

Subgroup	N. of studies	RD[a], MD[b], RR[c] (95% CI)	I2 (%)	P value I2	Range
Any respiratory symptoms in comprehensive SFL setting[a]					
Total	10	-0.19 (-0.26, -0.12)	70	0.001	(-0.18, 0.21)
Omitting two [76]	9	-0.21 (-0.27,-0.15)	41	0.10	
Any respiratory symptoms in partial SFL setting[a]					
Tota	4	-0.20 (-0.31, -0.08)	54	0.09	(-0.14, -0.25)
Omitting one [56]	3	-0.14 (-0.19, -0.10)	0	0.45	
Any sensory symptoms in comprehensive SFL setting[a]					
Total	9	-0.34 (-0.46, -0.22)	86	<0.001	(-0.31, -0.37)
FEV1 in comprehensive SFL setting[b]					
Total	3	0.10 (-0.04, 0.24)	87	<0.001	(-0.04, 0.13)
Asthma admissions in general population[c]					
Total	6	0.87 (0.81, 0.93)	78	<0.001	(0.85, 0.89)
Asthma admissions in children[c]					
Total	5	0.85 (0.79, 0.91)	87	<0.001	(0.82, 0.86)
Omitting one [59]	4	0.82 (0.79, 0.84)	0	0.77	
Asthma admissions in adults[c]					
Total	7	0.85 (0.73, 0.99)	65	0.009	(0.80, 0.90)
Omitting two [42] [48]	5	0.77 (0.70, 0.85)	31	0.22	
COPD admissions[c]					
Total	6	0.80 (0.63, 1.00)	96	<0.001	(0.73, 0.85)

Abbreviations: CI, confidence interval; COPD, chronic obstructive pulmonary disease; FEV1, forced expired volume in one second ; MD, mean difference; N, number; RD, risk difference; RR, risk ratio, SFL, smokefree legislation.

References:

42. Gaudreau K, Sanford CJ, Cheverie C, McClure C (2013) The Effect of a Smoking Ban on Hospitalization Rates for Cardiovascular and Respiratory Conditions in Prince Edward Island, Canada. PLoS ONE 8: e56102.

48. Humair J-P, Garin N, Gerstel E, Carballo S, Carballo D, Keller P-F, et al. (2014) Acute Respiratory and Cardiovascular Admissions after a Public Smoking Ban in Geneva, Switzerland. PLoS ONE 9(3): e90417. doi:10.1371/journal.pone.0090417.

56. Madureira J, Mendes A, Almeida S, Teixeira JP (2012) Positive impact of the Portuguese smoking law on respiratory health of restaurant workers. J Toxicol Environ Heal - Part A Curr Issues 75:776–787.

59. Millett C, Lee JT, Laverty AA, Glantz SA, Majeed A (2013) Hospital admissions for childhood asthma after smoke-free legislation in England. Pediatrics 131: e495–501.

76. Eagan TML, Hetland J, Aarø LE (2006) Decline in respiratory symptoms in service workers five months after a public smoking ban. Tob Control 15(3):242–246.
