# Supplementary material for: Smokefree legislation effects on respiratory and sensory disorders: A systematic review and meta-analysis
Source: PLoS One. 2017 Jul 31;12(7):e0181035. doi: 10.1371/journal.pone.0181035 (PMC5536320; doi:10.1371/journal.pone.0181035)
Supplement: S5 Table — (PDF) [file pone.0181035.s006.pdf]

S5 Table. Subgroup analysis by study design, risk of bias and follow-up time among studies relating any respiratory and sensory symptoms.

Subgroup	Number of studies	RD (95% CI)	I2 (%)	P value I2
Any respiratory symptoms in comprehensive SFL setting				
Total	10	-0.19 (-0.26, -0.12)	70	0.001
Type of study design				
Non experimental	9	-0.20(-0.28, -0.12)	73	<0.001
Quasi-experimental	1	-0.17 (-0.28, -0.05)	90	0.005
Risk of bias				
Low	6	-0.20 (-0.30, -0.10)	80	<0.001
Moderate	2	-0.23 (-.34, -0.11)	0	0.47
High	2	-0.16 (-0.26, -0.06)	0	0.33
Follow-up time				
<12 months	4	-0.25 (-0.42, -0.07)	88	<0.001
≥12 months	6	-0.16 (-0.21, -0.11)	0	0.64
Any respiratory symptoms in partial SFL setting				
Total	4	-0.20 (-0.31, -0.08)	54	0.09
Type of study design				
Non experimental	3	-0.19(-0.32, -0.05)	63	0.007
Quasi-experimental	1	-0.28 (-0.51, -0.05)		
Risk of bias				
Low	1	-0.14 (-0.19, -0.09)		
Moderate	2	-0.34 (-.50, -0.18)	0	0.51
High	1	-0.10 (-0.28, -0.08)		
Follow-up time				
<12 months	1	-0.10 (-0.28, 0.08)		
≥12 months	3	-0.24 (-0.41, -0.08)	68	0.05
Any sensory symptoms in comprehensive SFL setting				
Total	9	-0.34 (-0.46, -0.22)	86	<0.001
Type of study design				
Non experimental	8	-0.36(-0.49, -0.22)	87	<0.001
Quasi-experimental	1	-0.22 (-0.34, -0.11)		
Risk of bias				
Low	5	-0.36 (-0.54, -0.18)	91	<0.001
Moderate	2	-0.43 (-.57, -0.29)	36	0.21
High	2	-0.18 (-0.28, -0.08)	0	0.48
Follow-up time				
<12 months	3	-0.48 (-0.60, -0.36)	52	0.12
≥12 months	6	-0.27 (-0.39, -0.15)	81	<0.001

Abbreviations: CI, confidence interval; RD, risk difference; SFL, smokefree legislation.
