# Supplementary material for: Smokefree legislation effects on respiratory and sensory disorders: A systematic review and meta-analysis
Source: PLoS One. 2017 Jul 31;12(7):e0181035. doi: 10.1371/journal.pone.0181035 (PMC5536320; doi:10.1371/journal.pone.0181035)
Supplement: S6 Table — (PDF) [file pone.0181035.s007.pdf]

S7 Table. Subgroup analysis by study design, risk of bias and follow-up time among studies relating asthma and COPD admissions in comprehensive SFL setting.

Subgroup	Number of studies	RD (95% CI)	I2 (%)	P value I2
Asthma admissions in general population				
Total	6	0.87 (0.81, 0.93)	78	<0.001
Type of study design				
Non experimental	3	0.84(0.76, 0.93)	79	0.008
Quasi-experimental	3	0.90 (0.78, 1.03)	80	0.007
Risk of bias				
Low	2	0.89 (0.76, 1.03)	81	0.02
Moderate	3	0.82 (0.73, 0.92)	84	0.002
High	1	0.98 (0.85, 1.13)		
Follow-up time				
<24 months	2	0.87 (o.68, 1.10)	84	0.01
≥24 months	4	0.87 (0.81, 0.94)	80	0.002
Asthma admissions in children				
Total	5	0.85 (0.79, 0.91)	87	<0.001
Type of study design				
Non experimental	4	0.85(0.79, 0.91)	90	<0.001
Quasi-experimental	1	1.11 (0.63, 1.96)		
Risk of bias				
Low	4	0.85 (0.79, 0.92)	90	<0.001
Moderate	1	0.82 (0.71, 0.95)		
Asthma admissions in adults				
Total	7	0.85 (0.73, 0.99)	65	0.009
Type of study design				
Non experimental	6	0.80(0.71, 0.92)	54	0.005
Quasi-experimental	1	1.48 (0.90, 2.43)		
Risk of bias				
Low	3	0.95 (0.74, 1.22)	57	0.10
Moderate	4	0.79 (0.63, 0.98)	65	0.04
Follow-up time				
<24 months	1	1.17 (0.82, 1.76)		
≥24 months	6	0.81 (0.70, 0.93)	59	0.03
COPD admissions				
Total	6	0.80 (0.63, 1.00)	96	<0.001
Type of study design				
Non experimental	4	0.78(0.56, 1.09)	97	<0.001
Quasi-experimental	2	0.83 (0.74, 0.94)	40	0.20
Risk of bias				
Low	2	0.84 (0.73, 0.98)	61	0.11
Moderate	3	0.74 (0.47, 1.16)	96	<0.001
High	1	0.88 (0.78, 0.99)		
Follow-up time				
<24 months	2	0.61 (0.59, 0.63)	0	0.35
≥24 months	4	0.92 (0.79, 1.08)	80	0.002

Abbreviations: COPD, chronic obstructive pulmonary disease; CI, confidence interval; RD, risk difference; SFL, smokefree legislation.
