# Supplementary material for: Smokefree legislation effects on respiratory and sensory disorders: A systematic review and meta-analysis
Source: PLoS One. 2017 Jul 31;12(7):e0181035. doi: 10.1371/journal.pone.0181035 (PMC5536320; doi:10.1371/journal.pone.0181035)
Supplement: S1 Fig — Publication bias. (PDF) [file pone.0181035.s008.pdf]

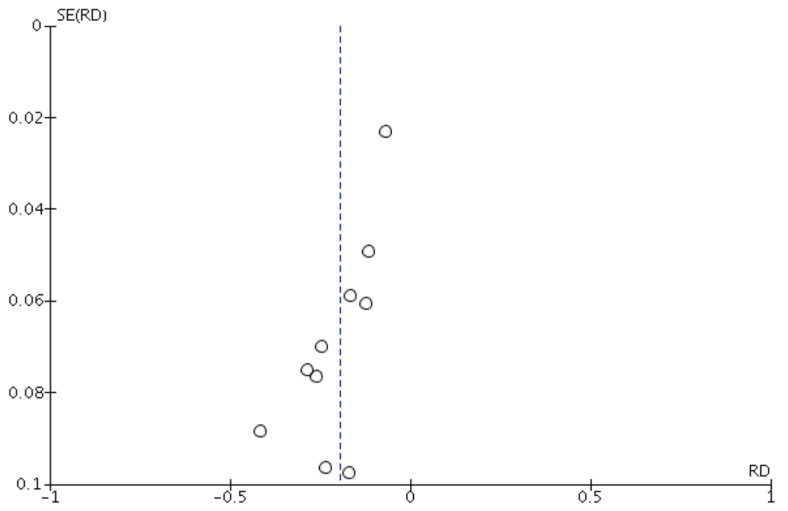

S6 Fig. Funnel plot of any respiratory symptom in comprehensive smokefree legislation setting. Publication bias
